# Proteomic profile of *pre - B2* lymphoblasts from children with acute lymphoblastic leukemia (ALL) in relation with the translocation (12; 21)

**DOI:** 10.1186/1559-0275-11-31

**Published:** 2014-08-01

**Authors:** Odile Costa, Pascale Schneider, Laurent Coquet, Philippe Chan, Dominique Penther, Elisabeth Legrand, Thierry Jouenne, Marc Vasse, Jean-Pierre Vannier

**Affiliations:** 1 Laboratoire MERCI, Faculté de Médecine et de Pharmacie de Rouen, 123 boulevard Gambetta, Rouen, Cedex 76183, France; 2 Service d’Immuno-Hématologie Onco-pédiatrique du CHRU de Rouen, Hôpital Charles Nicolle, Rouen 76031, France; 3 PISSARO Proteomic facility, (IRIB), U-Rouen, Mont Saint- Aignan, France; 4 CNRS UMR 6270, Team « Biofilms, Résistance, Interactions Cellules-Surfaces », U-Rouen, Mont Saint-Aignan, France; 5 Laboratoire de Cytogénétique, Centre Henri Becquerel, Rouen 76000, France

**Keywords:** Childhood ALL, Biomarkers, Calponin, OTUB1, Protein casein kinase CK2α and Ikaros

## Abstract

**Background:**

Until now, the major prognostic factors for pediatric acute lymphoblastic leukemia (ALL), age, white blood cell count and chromosomal alterations are initially taken into account for the risk stratification of patients. In the light of protein marker studies to classify subtypes of Acute Myeloblastic Leukemia efficiently, we have compared the lymphoblastes proteome in Childhood ALL in accordance with the presence of t(12;21), indicator of good prognosis, usually.

**Methods:**

Protein expression in pre-B2 lymphoblastic cells, collected from residual bone marrow cells after diagnostic procedures, was analyzed using two dimensional gel electrophoresis protocol. Protein spots whose average normalized volumes were statistically different in the two patients groups (n = 13; student *t* test p < 0.01), were excised. Tryptic peptides were then analyzed using a nano-LC1200 system coupled to a 6340 Ion Trap mass spectrometer equipped with a HPLC-chip cube interface. The tandem mass spectrometry peak lists extracted using the DataAnalysis program, were compared with the protein database Mascot Daemon.

**Results:**

We focused on twelve spots corresponding to sixteen identified candidate proteins among the 26 found differentially expressed (p ≤ 0.05) regarding the presence of t(12;21). Among over expressed proteins, two proteins were implicated in cellular growth arrest (*i.e.* calponine 2, p ≤ 0.001 and phosphatidylinositol transfer protein beta, p ≤ 0.001) in accordance with good prognosis, while two other proteins favored cell cycle proliferation (*i.e.* methionine adenosyl transferase 2β, p ≤ 0.005 and heterogeneous nuclear ribonucleo-proteins A2 p ≤ 0.01) and could therefore be good marker candidates of aggressiveness. Level of expression of proteasome subunit beta type-2 (p ≤ 0.01) and protein casein kinase 2α (p ≤ 0.01) which both favored apoptosis, deubiquitinating enzyme OTUB1 (p ≤ 0.05) and MLL septin-like fusion protein MSF-B, septin 9 i4 (p ≤ 0.01) were in accord with a good prognosis related to t(12;21) lymphoblasts.

**Conclusion:**

By drawing up the protein map of leukemic cells, these new data identified marker candidates of leukemic aggressiveness and new t(12;21) patients subgroups. These preliminary results will be in the near future confirmed by using a larger sample of pre-B2 childhood ALLs from national lymphoblastic cell collections.

## Background

The most common form of childhood cancer is the acute lymphoblastic leukemia (ALL), representing up to 30% of childhood cancers. The major prognostic factors, age and white blood cell count, are initially taken into account for the risk stratification of patients. Recurrent chromosomal alterations detectable by karyotyping, fluorescence in situ hybridization (FISH), or molecular techniques are found in 75% of childhood ALL and have important implications on prognosis [1]. The chromosomal rearrangement t(12;21) *ETV6-RUNX1* is associated with favorable outcome in B-ALL and much more frequent in childhood ALL (25% of B lineage ALL) [2]. The identification of genomic alterations is currently transforming our understanding of the genetic basis of leukemogenesis. Among these new genetic factors, alterations of the lymphoid transcription factor *IKAROS (IKZF1)* in B-ALL are remarkably associated with a poor outcome [3]. Though it is not the only example, only few other genetic alterations can be clearly associated with outcome in childhood ALL. This new insight on genomic profile of childhood ALL still has to be further explored. In the light of studies in which identified proteins, differentially expressed, could serve as markers to classify M1 and M2 subtypes of Acute Myeloblastic Leukemia, a correlation was also found between the accumulation of some proteins and therapeutic outcome and relapse time of the disease [4,5].

The present work relies on the global analysis of the protein expression of lymphoblastic cells and the identification of proteins as new biological markers, related to cytogenetic abnormalities. Therefore, we compared the whole proteome of bone marrow lymphoblasts collected by bone marrow aspiration during the diagnosis procedure. The proteome of each bone marrow lymphoblasts sample from pre-B2 t(12;21) ALLs, has been mapped using two dimensional poly acrylamide gel electrophoresis (2 DE) and compared to those from pre B2 ALLs without that translocation.

We identified twelve spots (corresponding to sixteen candidate proteins) whose average level was highly correlated either with the presence or the absence of t(12;21) *EV6-RUNX1* detected by cytogenetic analysis. Among the identified proteins two of them, protein kinase 2α (CK2α) and septin 9 i4 (*v4*) (SEPT9), are related to genes already identified and described in leukemogenesis, *i.e. IKAROS* and *MLL*, respectively [3]. CK2α is implicated in the phosphorylation of several proteins like phosphatase and tensin homolog (PTEN) and Ikaros acting on leukemia cell survival [6,7]. SEPT9 is implicated in cytokinesis and its role in tumorigenesis has been shown in various human tumors [8]. Beside these two proteins of interest we found other significantly differential amount of proteins related in some way to cell survival and already pointed out in several solid tumors. They could be related to several genes or pathways in relation with the regulation of the cell cycle, the apoptosis and also to the energetic metabolism of these very proliferative leukemic cells. Discussion debates about which protein might be a marker of good *versus* bad prognosis and which protein could be explored as a treatment target to defeat leukemogenesis.

## Results

### Patients

Among the thirteen patients (Table 1) with newly diagnosed B-ALL (pre-B2) which were included in this study, six children had an identified t(12;21) with an *ETV6-RUNX1* fusion transcript. The global outcome was good for both groups, 5/6 in the translocation group being alive in first complete remission (CR1) with a median follow-up, of 82 months (range 72–129), 6/7 were in CR1 in the group without t(12;21) (median follow-up 102.5 months; range 75–141). For both dead patients, death was directly related to the recurrence of ALL.

**Table 1:** Clinical and biological status of childhood pre-B2 ALL patients

**Group of ALL patients**	**Patients ID**	**Age at diagnosis (Months)**	**Peripheral blood lymphocytes (Giga/L)**	**Karyotype**	**Fusion transcripts**	**Outcome**
**With t(12; 21)**	**BACYO**	24	48,7	t(12; 21)	ETV6/RUNX1	CR1
	**GUEKE**	118	42,2	t(12; 21)	ETV6/RUNX1	CR1
	**LECRO**	76	5,4	t(12; 21)	ETV6/RUNX1	CR1
	**MARLE**	42	**166**	t(12; 21)	ETV6/RUNX1	CR1
	**PELAD**	50	20	t(12; 21)	ETV6/RUNX1	Dead*
	**RIFEM**	76	**216**	t(12; 21)	ETV6/RUNX1	CR1
**Other karyotype abnormalities**	**BOILE**	18	2,8	dic(9;20)	NA	CR1
	**CREBA**	**180**	11,6	mono 21	NA	CR1
	**DESNA**	13	15,5	rear9p	NA	CR1
	**JUGEL**	34	8,5	Hyperploidy	NA	CR1
	**LEMAL**	48	**56**	t(9; 22)	BCL/ABL1	CR1
	**MORLU**	**171**	**171**	t(9; 22)	BCL/ABL1	Dead*
	**ROSLO**	57	6,1	Hyperploidy	NA	CR1

The bold numbers indicates values higher the threshold of FRALLE 2000 protocol. CR1 = First complete remission; dic = dicentric; ID = Identification; mono = monosomy; NA = Not applicable; rear = Rearrangement. * = Directly related to the recurrence of ALL.

### Protein identifications

**Figure 1:**
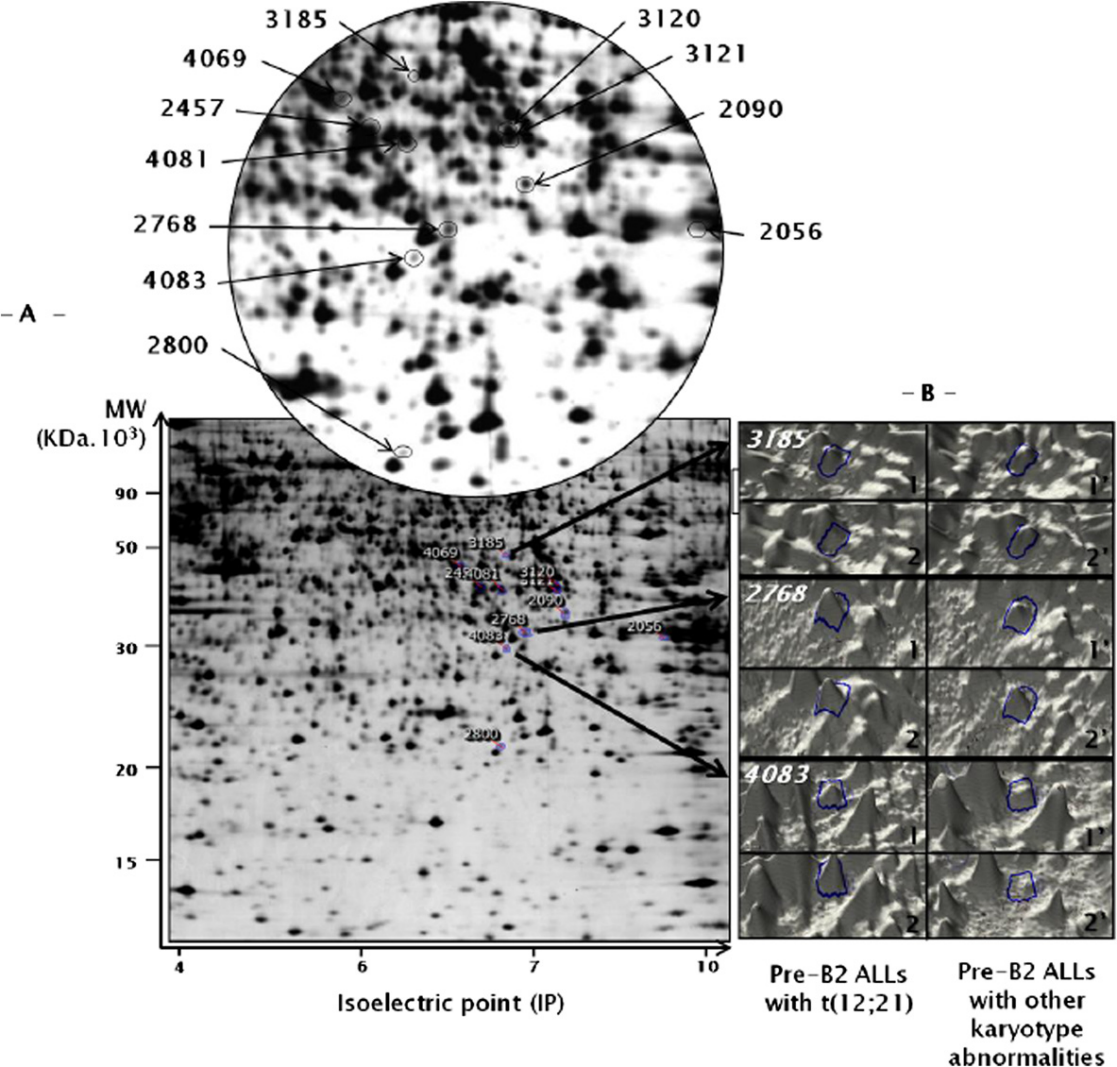
**Differential expression of proteins in bone marrow lymphoblastic cells from childhood pre-B ALL. – A –***Down*: Two-DE gel image of lymphoblastic cells proteins issuing from bone marrow. Numbers refer to the top eleven proteins which have been characterized and listed in Table 1b. *Up*: Magnification view of interesting zone **– B –** 3 D montage of the three top differentially expressed spot proteins (*i.e., 2-DE Batch* 2768, 3185 and 4083 on rank 1, 2 and 3, respectively). Only 2 individual spot images in each group (1 and 2 = Pre-B2 ALL with t(12;21), 1′ and 2′ = Pre-B2 ALL with other karyotype abnormalities) are shown.

**Figure 2:**
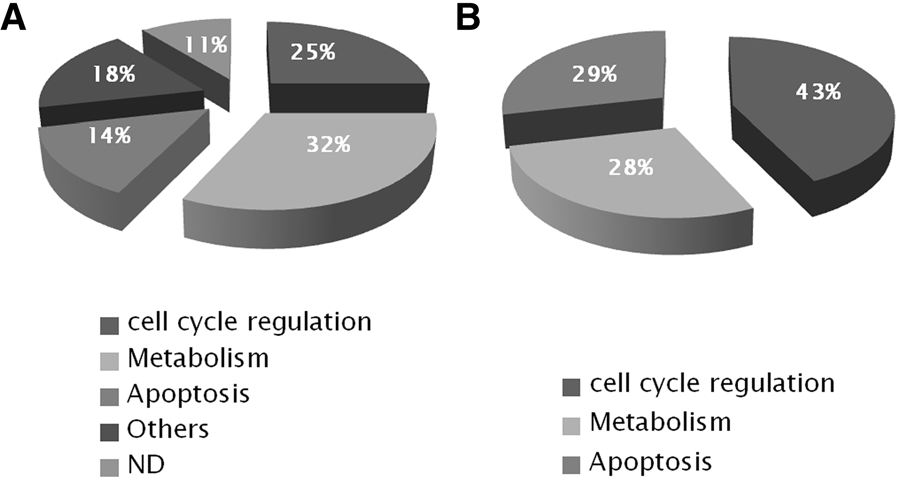
**Pie chart representing the principal protein’s functions with differential expression in lymphoblasts from pre-B2 ALL children, depending on the presence or absence of the translocation t(12; 21).** Proteins are characterized by LC-MSMS and Mascot score from 2-DE spots with: **A**- Differential expression with p ≤ 0.05 (n = 28); **B**- Differential expression with p ≤ 0.01 (n = 15). ND = none defined.

**Figure 3:**
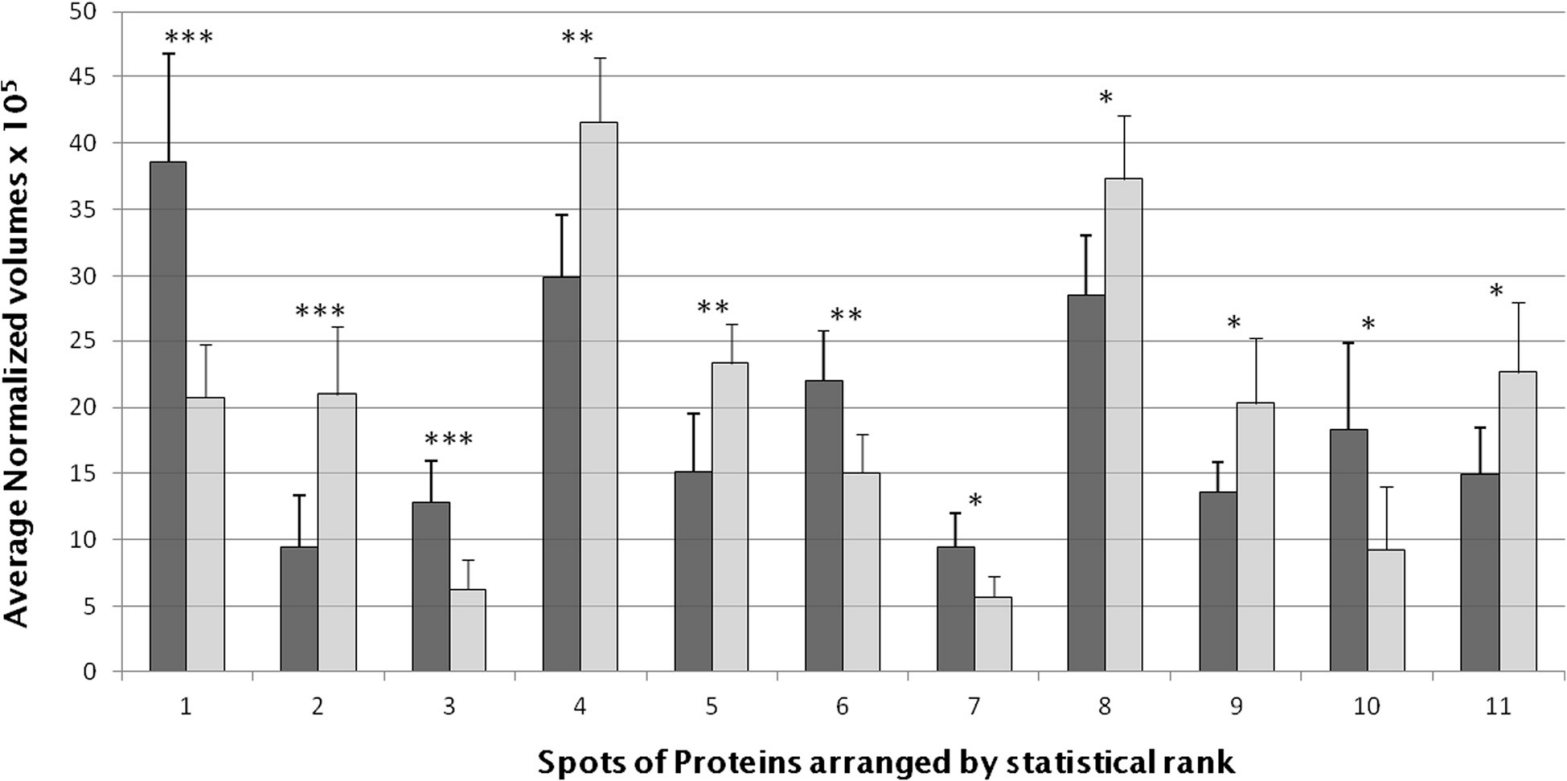
**Histogram of the average normalized volumes of the top eleven spots of protein with differential expression in lymphoblasts from pre-B2 ALL children, depending on the presence or absence of the translocation t(12;21).** ∎ = Pre-B2 ALLs with t(12;21), n = 6; □ = Pre-B2 ALLs with other karyotype abnormalities, n = 7. p ≤ 0.001***; p ≤ 0.005**; p ≤ 0.01*. Numbers are protein arranged by statistical rank, listed in Table 2.

**Figure 4:**
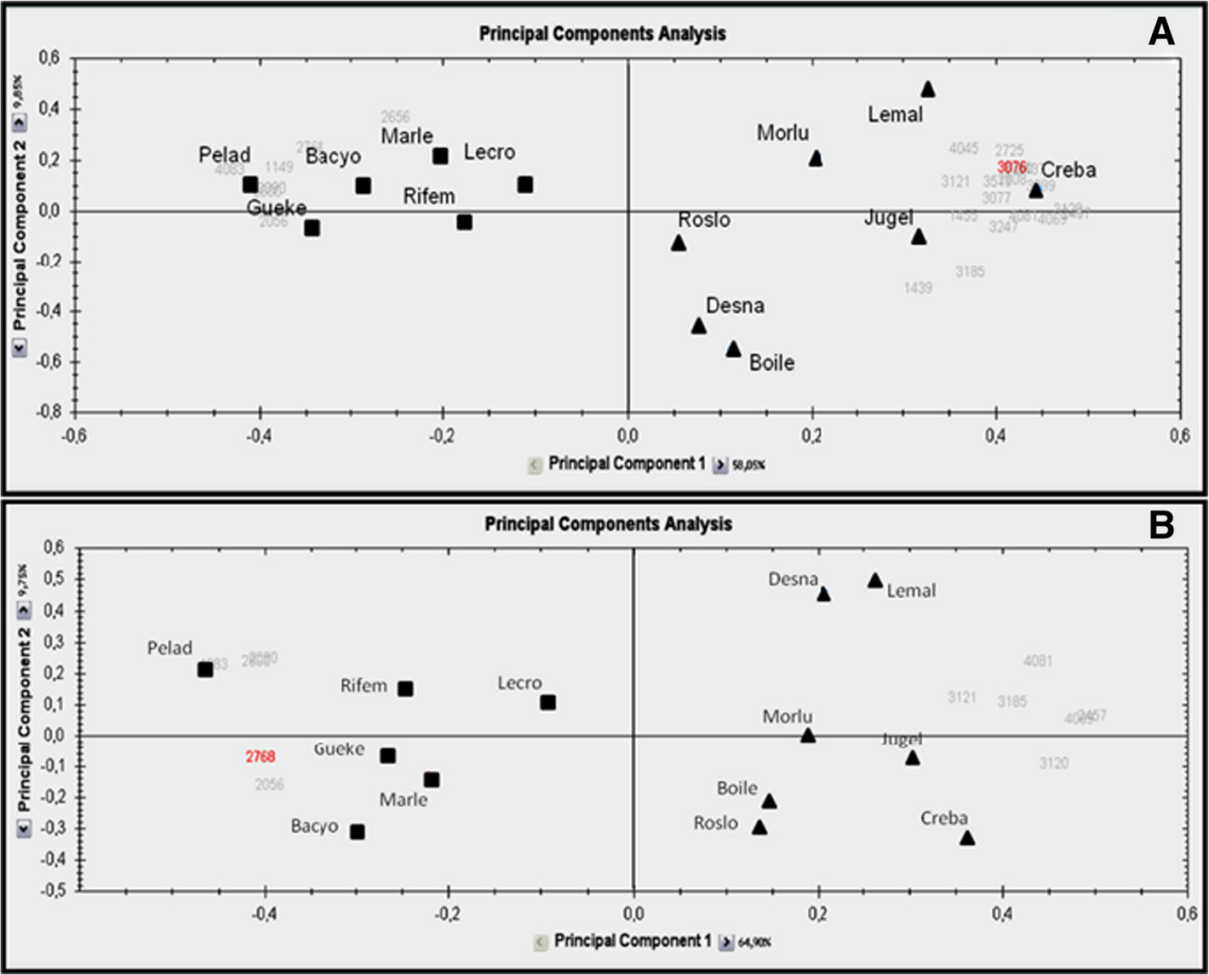
**Principal components analysis of spots with differential expression (student *t *test, t < 0.01), in lymphoblasts from pre-B2 ALL children, depending on the presence or absence of the translocation t(12; 21)**. Results were obtained with Progenesis SameSpotsV4. Left dotes (∎) are t(12;21) pre B2 ALLs patients (n = 6); Right dotes (▲) are other karyotype abnormalities in pre B2 ALLs patients (n = 7) (see comments below). **A**/ Spots differentially expressed with student *t* test value: p > 0.01 and p ≤ 0.05 (n = 15); **B**/ Spots differentially expressed with p ≤ 0.01(n = 11). Names close to dotes are identification badge of patients listed in Table 1.

A total of 4000 spots were detected on 2-DE maps (Figure 1). Protein maps were gathered with regard to the presence (n = 6) or the absence (n = 7) of t(12;21) (Table 1). Twenty six spots were found differently expressed in the two groups of patients (Table 2 for the top 12 spots; the whole spot informations can be seen in Online Additional file 1: Table S1) (student-*t* test, p ≤ 0.05). Repeatability assay was performed as indicated in *Patients, material and methods*, giving reliable results (see Online Additional file 1: Figure S1). Most of these proteins are involved in the cell cycle regulation (25%), metabolism (32%) and apoptosis (14%) (Figure 2A). The last ones (18%) are implicated in cytoskeleton organization, protein elongation or posttranslational events while 11% are not yet identified. However, among the 26 differentially expressed spots, only eleven spots (Figure 1A, see also Online Additional file 1: Figure S2 which located the last 15 spots), representing fifteen candidate proteins, were expressed with a sufficient reliable statistic power (student-*t* test, p ≤ 0.01, with a Power higher than 80%) in the two groups of patients (Figure 3). The average normalized volumes values and statistic ranks are listed Table 2. Five of them were expressed mostly in t(12;21) ALLs. The last seven spots were expressed mostly in the absence of this translocation. Corresponding proteins (Table 2, and Online Additional file 1: Table S1), identified by Mass Spectrometry with relevant Mascot score (more than 3 ions scores > 48) (see Online Additional file 1: Figure S3) were of great interest in terms of leukemogenesis since involved in mechanisms related to either the cell cycle regulation, or apoptosis, or energetic metabolism (*i.e.,* 43%, 29% or 28% of them, respectively) (Figure 2B). Principal components analysis (PCA) (see *Patients, materials and methods*) indicated that the amount of all the 26 spots of interest, when gathered together, could discriminate 2 groups of patient, correlating with the presence or absence of t(12;21) (Figure 4A,B).

**Table 2:** The classification of differentially expressed proteins in pre-B2 lymphoblastic cells from children with ALL

**Spots: statistic rank (2-DE batch)**	**Protein names [Homo sapiens]**	**NCBI and uniprot ID numbers**	**Fold changes/ALL t(12;21) *versus *** **others (average normalized volume +/- SD) 10**^**5**^	**Group with the upper average volume**	**Student -*t *** **test**	**Power (after q value)**
**1 (2768)**	CNN2	gi/49456619/ Q99439	**1.9/**(38.59 +/- 8.1 ***vs*** 20.76 +/- 4)	Pre - B2, t(12;21)	p ≤ 0.001	0.998
**2 (3185)**	CDSα	gi/32307132/ Q9Y697-1	**2.2/**(9.5 +/- 3.8 ***vs*** 20.98 +/- 5.2)	Other Pre - B2	p ≤ 0.001	0.985
**3 (4083)**	PITPβ	gi/6912594/ P48739	**2/(**12.8 +/- 3.1 ***vs*** 6.28 +/- 2.21)	Pre - B2, t(12;21)	p ≤ 0.001	0.983
**4 (2457)**	hnRNP-E1	gi/460771/ Q15365	**1.4/**(29.87 +/- 4.69 ***vs*** 41.57 +/- 4.88)	Other Pre -B2	p ≤ 0.005	0.976
	BUB3α	gi/4757880/ O43684				
**5 (4069)**	PDH-E1	gi/149242791/ P13804 -1	**1.5/**(15.16 +/- 4.33 ***vs*** 23.29 +/- 3.12)	Other Pre -B2	p ≤ 0.005	0.955
**6 (2090)**	MAT2β i1	gi/11034825/ Q9NZL9	**1.5/**(21.94 +/- 3.89 ***vs*** 15.06 +/- 2.91)	Pre - B2, t(12;21)	p ≤ 0.005	0.915
**7 (2800)**	PSMB2	gi/4506195/ B7Z478	**1.7/**(9.48 +/- 2.49 ***vs*** 5.69 +/- 1.63)	Pre - B2, t(12;21)	p ≤ 0.01	0.878
**8 (4081)**	CECR5	gi/14861834/ Q9BXW7	**1.3/**(28.51 +/- 4.48 ***vs*** 37.3 +/- 4.81)	Other Pre - B2	p ≤ 0.01	0.867
	BUB3α	gi/4757880/ O43684				
**9 (3121)**	CK2α	gi/4503095/ P68400	**1.5/**(13.55 +/- 2.36 ***vs*** 20.31 +/- 4.9)	Other Pre - B2	p ≤ 0.01	0.864
	SEPT9 i3	*gi/668381/* Q9UHD8-3				
**10 (2056)**	HnRNPA2	gi/4504447/ P22626-2	**2/**(18.28 +/- 6.56 ***vs*** 9.26 +/- 4.77)	Pre - B2, t(12;21)	p ≤ 0.01	0.854
**11 (3120)**	IVAD	gi/3212539/ P26440	**1.5/(**14.98 +/- 3.44 ***vs*** 22.64 +/- 5.34)	Other Pre B2	p ≤ 0.01	0.835
	FBA	gi/312137/ P05062				
	PSMB6	gi/1526426/ P62333				
**12 (2656)**	OTUB1	gi/6841176/ Q96FW1	**1.8**/(58.54 +/- 19.8 ***vs*** (32.01 +/- 14.53)	Pre - B2, t(12;21)	p ≤ 0.05	0.787

Two-dimensional gel electrophoresis (2-DE) Batches are located Figure 1A (in case of the top eleven) and Additional file 1: Figure S2 (for the following 15th). In column’ protein names, R1, R2 and R3 are the Mascot rank based on the whole ionic scores detected (upper or equal to 48). Power analysis is performed independently for each spot, taking into consideration the sample size and variance expression value (Progenesis SameSpots V4). CNN2 = calponin-2; CDSα = cysteine desulfurase, mitochondrial isoform a; PITPβ = phosphatidyl- inositol transfer protein beta isoform; BUB3a = mitotic checkpoint protein BUB3 isoform a; PDH E1 = chain A, pyruvate dehydrogenase E1 S264e Variant; MAT2β = methionine adenosyl transferase 2, subunit beta, isoform 1; PSMB2 = proteasome subunit beta type-2, isoform 1; CECR5 = cat eye syndrome critical region protein 5 isoform 2; CK2α = casein kinase II subunit alpha isoform a; SEPT9_i3 = MLL septin-like fusion protein MSF-B; hnRNPA2 = heterogeneous nuclear ribonucleo- proteins A2; IVAD = Chain A, isovaleryl-Coa dehydrogenase; FBA = fructose bisphosphate aldolase; PSMB6 = proteasome subunit 6 (p42); OTUB1 = deubiquitinating enzyme OTUB1.

Among the proteins involved in cell cycle regulation, are the calponin-2 (CNN2) and methionine adenosyltransferase 2beta (MAT2β) variant 1, which are highly expressed in ALL with t(12;21), usually associated to good prognosis (p < 0.001, with a power of 0.998; p < 0.005, with a power of 0.985, respectively) (see Table 2)*.* On the contrary hnRNP-E1, mitotic checkpoint protein BUB3α (or BUB3α) or MLL septin-like fusion protein MSF-B (SEPT9_i4) were weakly expressed in ALL with t(12;21), (p < 0.005).

Among proteins involved in apoptosis, phosphatidylinositol transfer proteins beta (PITPβ) was highly expressed in ALL with t(12;21), while the casein kinase 2 alpha (CK2α) protein was weakly expressed in this group of patients (p < 0.001 and p < 0.01, respectively). Proteasome subunit beta 2 (PSMB2), one of the two proteins of the PSM which we found, was predominant in ALL with t(12;21) (p < 0.01) while PSM beta 6 (or P42 *i.e.,* PSMB6) predominated in the other group of pre-B2 ALL (p < 0.01)*.* Interestingly, HSPC263 (*i.e.,* OTUB1), a deubiquitinating enzyme was slightly over expressed in t(12;21) ALLs, in the rank just after the first group of eleven spots described (Table 2).

The other proteins which were implicated in cellular metabolic pathways (*i.e.,* cystein desulfurase alpha (CDSα), pyruvate deshydrogenase E1 (PDH-E1), cat eye syndrome critical region protein 5 (CECR5), isovaleryl CoA deshydrogenase (IVAD) and fructose biphosphate aldolase (FBA)) were all together predominant in the group of pre-B2 ALL without t(12;21) (p < 0.005, p < 0.005, p < 0.01, p < 0.01, p < 0.01, respectively). CECR5 has not yet been described in lymphoblasts.

## Discussion

Little is known about proteomic profiling in childhood ALL. Recently, protein expression was compared in leukemia cell lines and in patients before (*i.e.,* at the diagnostic day) and after 7 days (D8) of prednisolone treatment [9]. The results focused on two proteins differentially expressed: the proliferating cell nuclear antigen, which plays important roles during DNA replication and repair and the voltage-dependent anion channel which participates in the formation of the permeability transition pore complex, are responsible for the release of mitochondrial products that trigger apoptosis [10]. All these data confirmed the interest of proteomics in hematology malignancy.

The aim of the present work was to identify proteins that could be related to a good prognosis, often associated to the translocation t(12;21) in childhood ALL. Thus, we pointed out differences in amounts of some proteins expressed in t(12;21) ALLs and non t(12;21) ALLs. These proteins have been classified regarding their biological activity. We discussed the relevance of these proteins as diagnostic markers and/or targets for leukemia therapy.

### Proteins involved in cell cycle regulation

Some proteins of this group are involved in the cell cycle regulation (e.g. CNN2 the neutral isoform which belongs to a family of actin filament-associated proteins). CNN2 has been shown to inhibit cell proliferation, suggesting its regulatory role in cytokinetic activities [11]. CNN2 is also known to activate PKC phosphorylation, a key mechanism that mediates growth arrest in tumor cell lines [12]. Its increased or reduced expression has been associated in solid tumors with good or poor prognosis, respectively [13]*. So, the over expression of CNN2 here observed in t(12;21) ALLs, could reduce the cell proliferation and could be related to the good prognosis usually associated to this translocation* (Table 3)*.*

**Table 3:** Concluding remarks and future directions

**Expression observed in t(12;21) ALLs**	**Major expected cellular effects**	**Comments**	**Future directions**
Over expression of CNN2	Mediates cellular growth arrest	In favor of good prognosis associated to t(12;21)	Is CNN2 a marker of good prognosis? It has to be confirmed with a largest group of patients
Over expression of MAT-2β	Favors the cell proliferation with anti-apoptotic effect	Would be a marker of cancer aggressiveness	Would be explore as a target of chemotherapy
Over expression of hnRNPA2	Favors the cell proliferation	Would be a marker of cancer aggressiveness	Would be explore as a target of chemotherapy
Over expression of PITPβ	Slow down the cell proliferation	Is it a marker of good prognosis?	Exploration of the balance PITPα/β
Under expression of BUB3	Avoid slippage of mitosis	Over expression in non t(12;21) could lead to hypo/hyperpolyploid cells	Mitoses analysis
Under expression of hnRNPE2	Favors apoptosis	In favor of good prognosis associated to t(12;21)	Exploration of the balance between hnRNPE2, PSMB2 and PSMB6 and/or checkup the apoptosis pathways
Over expression of PSMB2	Favors apoptosis		
Under expression of PSMB6	Hinders apoptosis	In favor of leukogenesis	
Under expression of CK2α	Slow down the proliferation by apoptotic effect	In favor of good prognosis associated to t(12;21)	Both expressions in combination with Ikaros (*Ikzf1*) phosphorylation and degradation ought to be clarified precisely
Over expression of HSPC263(OTUB1)	Favors deubiquitination	Does it prevent Ikaros (*Ikzf1*) degradation?	
Under expression of metabolism pathway proteins (*i.e.*, CDSα; CECR5; PDH; IVAD; IDH; Electron transfer flavoprotein)	Sign the level of energetic consumption	In favor of good prognosis associated to t(12;21)	Checkup lymphoblasts metabolism pathway: High amount would be in accord with cancer aggressiveness

The MAT2β subunit is the second protein of this group. It was found over expressed in t(12;21) ALLs. MAT2 is implicated in the methylation protein pathway (*i.e.* trans-methylation, trans-sulfuration, and polyamine synthesis) and acts as a transcriptional co-repressor of the oncogene MafK [14,15]. Accordingly, MAT2 activity is constitutively expressed in stimulated and actively dividing T cells, as well as T leukemic cells [16]. The subunit MAT2β, which is catalytically inactive, is the regulatory subunit of the isozyme [15,17]. When expression of MAT2β is successfully silenced, excessive apoptosis and growth slowdown is observed in Jurkat leukemic T cell [17,18]. Recently MAT2β v1 (here characterized), has been identified as an anti-apoptotic component through Sirtuin 1 signaling [19]. *Therefore, the over expression of MAT2β in t(12;21) ALLs cannot be considered as a good prognostic factor. It seems to have the opposite effect on cell proliferation when compared to CNN2. MAT-2β has to be evaluated as a possible target to improve prognosis in the t(12;21) ALLs* (Table 3)*.*

HnRNPA2 functions as telomeric capping factor protecting telomeric DNA *in vivo* against nuclease digestion by recruiting the essential RNA subunit (hTR) to the telomere [20]. RNAi, reduction of hnRNPA1 and hnRNPA2 significantly reduced the proliferation rate of Colo 16 cells, suggesting that these hnRNP members may be classified as oncogenes [21]. *HnRNPA2 is found overexpressed in t(12,21)ALLs and thus would contribute to favor the pre - B2 proliferation and would be a marker of leukemogenesis* (Table 3)*.*

The three last proteins included in this group (*i.e.* hnRNP-E1, BUB3α and SEPT9_i4) are under expressed in t(12;21) ALLs.

HnRNPE1 is a member of the RNA binding protein family which includes important mitotic regulators and effectors, having multiple functions in hematopoietic cells. It is involved in controlling stem cell proliferation and differentiation [21,22]. Particularly, acting as “chaperone” molecule on the RNA, hnRNPs E1 functions as crucial modulator of mRNA stability, and translation in hematopoietic cell differentiation [23]. It stimulates the translation of c-myc and Bag-1, having an anti-apoptotic effect on cancer cells [21,24]. The accumulation of hnRNPE1 in lymphoblastes could induce an anti-apoptotic effect, worsening the disease. *The under expression of hnRNPE2 observed in t(12;21) ALLs would contribute to apoptosis in these leukemic cells* (Table 3)*.*

The BUB3α is one of the components of the spindle assembly checkpoint (SAC) which interacts with BUB1 and leads to delay the cell cycle progression [25]. However, in conditions where the SAC is kept active, some cells escape from mitosis, resulting in polyploid cells [25]. *Its over expression in non t(12;21) ALLs could contribute to the slippage of mitosis toward a hypo/polyploid cells* (Table 3)*.*

SEPT9_i4 is an isoform of the GTP binding septin family, involved in cytokinesis. Over expression of the short isoform SEPT9_i4 disturbs septin interactions and cellular motility which can be related to neoplasic associated phenotypes [26]. SEPT9_i4 over expressing cells have enhanced survival in the presence of clinically relevant microtubule acting drugs. Its over expression in several cancers may be clinically relevant with a contribution of drug resistance forms of cancer [27]. This i4 variant is the COOH terminal sequence of all the isoformes [28]. Thereby it may be difficult to reach it as a specific target. By introducing mutations that prevent SEPT9 self - association at the N- and C-*termini*, it has been shown that septin filament disruption causes defects in the late stages of cell division before abscission, similar to these observed upon SEPT9 depletion [28].

### Proteins involved in apoptosis

Among proteins which are principally implicated in apoptotic pathways and may have a prognostic value (*i.e.,* PITPβ, CK2α, and PSMB2, PSMB6), phosphatidylinositol transfer proteins beta (PITPβ) and CK2α have an impact on Pl3K pathway.

PITPβ was highly expressed in the group of t(12; 21) ALLs. Physiologically, PITPβ is poorly expressed and favors the activation of a golgian PI3K [29]. PITPβ accumulation, by modifying the balance PITPα/β, induces a decrease of proliferation and an increase of apoptosis [30]. *The exploration of the balance PITPα/β in ALLs may be a useful biological marker.*

On the contrary, CK2α was under expressed in t(12; 21) ALLs. CK2α disturbs the PI3K pathway by phosphorylating PTEN and AKT, thereby favoring cell proliferation by an anti-apoptotic effect [7,31]. The over expression of CK2α has been shown to increase the degradation of Ikaros protein (*i.e.,* a tumor suppressor in ALL) *via* the ubiquitin pathway [3,32,33]. Interestingly in the t(12, 21)ALLs, not only CK2α is under expressed but OTUB1, a deubiquitinating enzyme, is found slightly over expressed (see Table 2, rank 12) [34]. OTUB1 hydrolase can specifically remove ‘Lys-48”-linked conjugated ubiquitin from proteins and plays an important regulatory role at the level of protein turnover by preventing degradation. It has recently been identified as a novel p53 regulator [35]. *All together this could result in an preservation of Ikaros functions and would contribute to the good prognostic claimed for t(12;21) ALLs.*

The two other proteins implicated in apoptosis (*i.e.*, PSMB2 and PSMB6) are constitutive of the proteasome. The proteasome is responsible for the degradation of intracellular proteins involved in cell cycle control and regulation of apoptosis, including the tumor suppressor protein p53 and specific cyclin dependent kinases [36]. At this time we cannot explain why PSMB2 is predominant in t(12;21) ALLs, while PSMB6 is weakly expressed. New therapies against tumor growth and development have an anti-ubiquitin-proteasome pathway effect [36].

Two pathways in which most of these proteins (*i.e.,* involved in apoptosis and also BUB3) were pointed out with Protein Center Software (Thermo Scientific ProteinCenter Software). The first one (Raw p-value, p < 6.98 10^-7^ and false discovery date (FDR), p < 5.41 10^-4^) is the negative regulation of protein ubiquitination (*i.e.,* BUB3, OTUB1, PSMB6 and PSMB2). The second one (Raw p-value: p < 1.8 10^-5^ and FDR: p < 1.5 10^-3^) is the negative regulation of mitotic cell cycle phase transition (*i.e.,* BUB3, CK2a, PSMB6 and PSMB2). Both of them involved the same proteins. However, OTUB1 and CK2α exhibit an opposite effect on ubiquitination, CK2 acting indirectly on cell cycle progression.

### Proteins involved in metabolic pathway

Proteins of the third group implicated in metabolic pathway (*i.e.,* CDSα, PDH-E1, IVAD and FBA) are predominant in pre B2 ALLs without t(12; 21). CDS, the second protein at the statistical average rank, is involved in cellular iron homeostasis and in the production of selenoproteins which have antioxidant functions and possibly cancer-protective effects [37]. IVAD and FBA are involved in glucose metabolism [38]. FBA participates to ATP synthesis in the glycolytic glycol pathway in cancer cells [39]. Interestingly, increased glycolytic rate in ALL is directly related to glucocorticoid resistance in primary leukemic cells of pediatric ALL patients [40]. The similarity of CECR5 to yeast phosphatidyl synthases suggests that it is an enzyme involved in fatty acid metabolism, possibly in the production/processing of membrane phospholipids [41]. *Its implication in ALLs has to be explored* (Table 3)*.*

### Conclusion and future directions

Through this protein profile of children with or without t(12;21), several proteins of interest emerge, defining a “protein-map” associated to some subgroups of patients with particular features. The correlation between the proteins expression and the t(12;21) or its fusion transcript *ETV6-RUNX1* still has to be confirmed. Nevertheless, this new approach for identification and classification of patient subgroups could lead to interesting therapeutic target. At the fundamental level, the identification of the biological pathway regulated by *RUNX1* is also of importance to define the role of these proteins in leukemogenesis. Indeed, this is well documented for the fusion transcripts but rare or inexistent at the level of proteins translation or modulation [42]. Altogether, this proteomic analysis focuses on major proteins (Table 3) playing important roles in leukemogenesis. At the present time, the difficulty is to conclude on the order of importance of the expression of each protein implication in the same way (*i.e.* cellular proliferation or apoptosis). All lymphoblasts studied are coming from ALLs and so are leukemic cells. Consequently, it is not conflicting to find CNN2 and PITPB over expressed, put on the brake of cell growth and while MAT-2β or HnRPA2 also over expressed in t(12, 21) ALLs speed up the cell cycle. Future work will be devoted to study a larger panel of patients, and to confirm the proteins CNN2, protein CK2α and OTUB1 as good candidates markers of prognosis and if the some other proteins could be targeted by chemotherapy. The monitoring of the amount of these proteins following the induction treatment could also indicate its signaling pathway efficiency [43]. At the same time, the metabolism pathway check-up would inform on the level of energetic consumption linked to cancer aggressiveness. Another interesting question concerns the impact of the expression of CK2α [6,7] and, possibly, OTUB1, on degrading Ikaros. Different ways of CK2 inhibition by small-molecule inhibitors have already been suggested as therapeutic tools for several cancers [44]. CK2 acts on Pl3K pathway by phosphorylating PTEN thereby leading to leukemia cell survival. It would be interesting to test *in vitro* inhibitors of CK2 and to compare their effect to inhibitors targeting PI3K/PKB [45]. Modulators or activators of OTUB1 would be also useful tools to control the ubiquitination/deubiquitination of proteins which slow down leukemogenesis (*i.e.*, Ikaros, particularly) [46].

## Patients, material and methods

### Patients

Thirteen children with newly diagnosed B-ALL are included in this study. Analyses were carried out on residual bone marrow cells (BMC) after diagnostic procedures, in agreement with the requirements of the ethical committee of the Rouen University Hospital and the French ethical laws [47]. All patients were included in the French protocol for childhood ALL (FRALLE 2000) [48]. Patients were classified in two different groups according to the presence or absence of a translocation t(12; 21).

### Cytogenetic and molecular analysis

Chromosome and FISH analysis were performed on unstimulated bone marrow and/or peripheral blood cells cultures using EV6-RUNX1 Abbott dual color DNA probes. EV6-RUNX1 fusion gene transcript is studied by real time –polymerase chain reaction as described in Biomed -1 concerted action’s report [49]*.*

### Cell preparation

Leukaemia cells from BMC were isolated by Ficoll-Hypaque (Eurobio, Les Ulis, France) density gradient centrifugation and stored at -80°C [50]. A Human pre-B-ALL line, Nalm 6, was grown as indicated by DSMZ. (DSMZ, Braunschweig).

### Two-dimensional electrophoresis (2-DE) of proteins

Proteins were extracted from dounce homogeneized cell pellets double washed in RPMI 1640, according to the protocol of the 2-D Clean-Up kit (GE Healthcare). The isoelectric focusing was performed by using dried 18-cm Immobiline pH 3–11NL (IPG) Strip rehydrated with 20 mM DeStreack IPG buffer pH 3–11, (GE Healthcare). Fifty μg of protein samples (or 300 μg for preparative 2-DE protocol) were loaded in anodic cup and proteins were focused using Ettan IPGphor 3 (pI 3–11 program). After equilibration, strips were sealed on the top of poly-acrylamide gel cassette precasted with the automate 2D Optimizer (Nextgen) (i.e. Sigmoid curve 6% to 18% gradient of Acrylamide-bis Acrylamide (37.5:1). 2-DE was carried out (500 V, 40 mA, 4 Hrs) in Ettan Dalt six Large Vertical System as described previously [51].

### Gel staining and image analysis

Analytical Gels were silver stained using PlusOne Staining kit, protein (GE Healthcare Lifescience) based on a simplified method. For preparative scale, the gels were stained by colloidal Coomassie blue G-250 (Sigma Chemical Co). After coloration, gels were scanned and quantified by computing scanning densitometry (ImagScanner III densitometer-GE Healthcare). A total of 4000 protein spots were discriminated on 2-DE (*i.e.,* whose the volume was up than 300,000). All gel images were normalized to the total valid spot volume of each gel and included for statistical group matches as described below.

### Global statistical analysis

Statistical analysis was carried out with Progenesis SameSpotsV4 [52]. Before “Progenesis software” analysis, the first step of analysis was to check the repeatability of our analysis by comparing randomly spots average volume in three gels per patient (see Additional file 1: Figure S1A). We indeed observed that artifactual results could be obtained when superposition of too many gels (each of them exhibiting about 4000 spots) was performed in the same analysis. That is the reason why in the next step, one representative of the three gels per patients was selected for gel images alignment (see Additional file 1: Figure S1B). Then statistic analysis has been performed with “Progenesis software” and the results are represented Figure 3, pointing out the eleven spots of the main area (seen in Figure 1). In the last step, we returned on our analysis, pointed out these eleven spots of the main area and compared the three protein maps for each thirteen patients (see Additional file 1: Figure S1B).

Gel spots whose intensity change was greater than 30% (student *t* test; p < 0.01) and exhibiting a power higher than 0.8 were claimed statistically relevant (Progenesis SameSpots V4). Therefore, multivariable statistical analysis was performed on data matrix of 15/or 12 rows (*i.e.,* spots proteins; Figure 4A and B, respectively) and 2 columns (patients with t(12;21) and other patients). Automatic correlation and hierarchical clustering of protein spot intensities and Principal component analysis (PCA) was applied, using Progenesis Samespots software, to analyse the similarity of protein patterns among gels and the expression profiles of protein spots fulfilling the above criteria. PCA of raw data (*i.e.,* spot volume values, average over 13 patients) allowed to discriminate two Principal Components (PC1 and PC2) which together explained 67.9% or 74.65% of the variance (Figure 4A and B, respectively). The PC1 accounted for up to 67.9% of the variance and was related to the diagnostic, each being distributed along PC1.

### Mass spectrometry identification

Spots of interest were excised and trypsin digestion was performed as previously described [51]. Peptide were then analyzed using a nano-LC1200 system coupled to a 6340 Ion Trap mass spectrometer equipped with a HPLC-chip cube interface (Agilent Technologies, Massy, France). The tandem mass spectrometry peak lists were extracted using the DataAnalysis program (version 3.4, Bruker Daltonic) and compared with the protein database using Mascot Daemon (version 2.1.3). The determination of at least three peptide sequences with a Mascot Ion Score over 48 allowed a satisfactory identification of the protein.

## Abbreviations

2-DE: Two-dimensional polyacrylamide gel electrophoresis; ALL: Acute lymphoblastic leukemia; CNN2: h2-calponin; MAT2Bβ: Methionine adenosyltransferase 2β; hnRNP-E1: Heterogeneous nuclear ribonucleoprotein-E1; BUB3α: Mitotic checkpoint protein BUB3 isoform a; MSF-B: SEPT9_i4, MLL septin-like fusion protein; PITPβ: Phosphatidylinositol transfer proteins beta; CK2α: Casein kinase 2alpha; PSMB6: Proteasome subunits beta 2, PSMB2, proteasome subunits beta 6 (p42); CDSα: Cystein desulfurase mitochondrial isoform α; PDH: Pyruvate dehydrogenase; IVAD: Isovaleryl CoA dehydrogenase.

## Competing interests

The authors declare that they have no competing interests.

Online Supplementary informations are available at http://www.haematologica.org.

## Authors’ contributions

OC, PhD, MCU, performed and designed research, analyzed data, and wrote the manuscript; PS, MD, PUPH, is pediatric hematologist and wrote the manuscript; EL, Technician, keep growing cell lines, DP, MD, performed cytogenetic analysis, LC, PhD, RI, and PC, PhD, RI, performed mass spectrometry research, TJ, PhD, PU, Director of PISSARO Proteomic facility; MV, PUPH and J-PV, MD, PUPH contributed vital reagents. All authors read and approved the final manuscript.

## Supplementary Material

Additional file 1: Table S1 Classification of differentially expressed proteins in pre-B2 lymphoblastic cells from children with ALL. 2-DE Batches are located Figure 1b. R1, R2 and R3 (In protein names column) are the Mascot rank basis on the whole ionic scores (IS) detected (Here only IS, listed in Additional file 1: Figure S3, upper or equal to 48 are taken in consideration); Power analysis was performed independently for each spot, taking into consideration the sample size and variance expression value (Progenesis SameSpots V4). **Figure S1.** Normalized volume of silver stained spots from two dimensional gels: A- Average normalized volume realized from 9 patients which were chosen randomly. Gels were performed in triplicate (bars: SD, n=3). Spot A to spot L were chosen randomly to valid the method. B- Normalized volume realized from 13 patients included in final statistical analysis (referred in the main manuscript Figure 3), focusing on the 11 first spots discussed in the main document. Values of three 2D maps for each ALL patients with t(12;21) (Ba), or ALL patients without this translocation (Bb). **Figure S2.** Spot localization on 2-DE gels: Only spots submitted with success to the student t test values (p>0.01 and p≤ 0.05) are numbered. Numbers correspond to protein spots listed in Table 2. **Figure S3.** Mascot research results for the first eleven spot proteins ordered by statistic rank (Progenesis SameSpotsV4 software –see Additional file 1: Table S1). For each identified trypsic peptide, individual ions scores up to 48 indicate identity or extensive homology (p<0.05). Matched peptides are shown in bold red. Click here for file

## References

[B1] Mullighan CG The molecular genetic makeup of acute lymphoblastic leukemia Hematology Am Soc Hematol Educ Program 2012 11 389 396 Review 23233609 10.1182/asheducation-2012.1.389

[B2] Romana SP Poirel H Leconiat M Flexor MA Mauchauffé M Jonveaux P Macintyre EA Berger R Bernard OA High frequency of t(12;21) in childhood B-lineage acute lymphoblastic leukemia Blood 1995 11 4263 4269 7492786

[B3] Li Z Song C Ouyang H Lai L Payne KJ Dovat S Cell cycle-specific function of Ikaros in human leukemia Pediatr Blood Cancer 2012 11 69 76 22106042 10.1002/pbc.23406 PMC3292658

[B4] Kornblau SM Tibes R Qiu YH Chen W Kantarjian HM Andreeff M Coombes KR Mills GB Functional proteomic profiling of AML predicts response and survival Blood 2009 11 154 164 18840713 10.1182/blood-2007-10-119438 PMC2951831

[B5] Luczak M Kaźmierczak M Handschuh L Lewandowski K Komarnicki M Figlerowicz M Comparative proteome analysis of acute myeloid leukemia with and without maturation J Proteomics 2012 11 5734 5748 22850270 10.1016/j.jprot.2012.07.030

[B6] Martins LR Lúcio P Silva MC Gameiro P Silva MG Barata JT On CK2 regulation of chronic lymphocytic leukemia cell viability Mol Cell Biochem 2011 11 51 55 21750986 10.1007/s11010-011-0947-6

[B7] Barata JT The impact of PTEN regulation by CK2 on PI3K-dependent signaling and leukemia cell survival Adv Enzyme Regul 2011 11 37 49 21035501 10.1016/j.advenzreg.2010.09.012

[B8] Scott M Hyland PL McGregor G Hillan KJ Russell SE Hall PA Multimodality expression profiling shows SEPT9 to be overexpressed in a wide range of human tumours Oncogene 2005 11 4688 4700 15782116 10.1038/sj.onc.1208574

[B9] Jiang N Kham SK Koh GS Suang Lim JY Ariffin H Chew FT Yeoh AE Identification of prognostic protein biomarkers in childhood acute lymphoblastic leukemia (ALL) J Proteomics 2011 11 843 857 21396490 10.1016/j.jprot.2011.02.034

[B10] Gazy I Kupiec M The importance of being modified: PCNA modification and DNA damage response Cell Cycle 2012 11 2620 2623 22732495 10.4161/cc.20626

[B11] Hossain MM Hwang DY Huang QQ Sasaki Y Jin JP Developmentally regulated expression of calponin isoforms and the effect of h2-calponin on cell proliferation Am J Physiol Cell Physiol 2003 11 C156 C167 12388067 10.1152/ajpcell.00233.2002

[B12] Wu KC Jin JP Calponin in non-muscle cells Cell Biochem Biophys 2008 11 139 148 18946636 10.1007/s12013-008-9031-6

[B13] Yamamura H Yoshikawa H Tatsuta M Akedo H Takahashi K Expression of the smooth muscle calponin gene in human osteosarcoma and its possible association with prognosis Int J Cancer 1998 11 245 250 9645345 10.1002/(sici)1097-0215(19980619)79:3<245::aid-ijc6>3.0.co;2-p

[B14] Katoh Y Ikura T Hoshikawa Y Tashiro S Ito T Ohta M Kera Y Noda T Igarashi K Methionine adenosyltransferase II serves as a transcriptional corepressor of Maf oncoprotein Mol Cell 2011 11 554 566 21362551 10.1016/j.molcel.2011.02.018

[B15] Nordgren KK Peng Y Pelleymounter LL Moon I Abo R Feng Q Eckloff B Yee VC Wieben E Weinshilboum RM Methionine adenosyltransferase 2A/2B and methylation: gene sequence variation and functional genomics Drug Metab Dispos 2011 11 2135 2147 21813468 10.1124/dmd.111.040857 PMC3198898

[B16] Wang Q Liu QY Liu ZS Qian Q Sun Q Pan DY Inhibition of hepatocelluar carcinoma MAT2A and MAT2beta gene expressions by single and dual small interfering RNA J Exp Clin Cancer Res 2008 11 72 80 19025580 10.1186/1756-9966-27-72 PMC2613873

[B17] Jani TS Gobejishvili L Hote PT Barve AS Joshi-Barve S Kharebava G Suttles J Chen T McClain CJ Barve S Inhibition of methionine adenosyltransferase II induces FasL expression, Fas-DISC formation and caspase-8-dependent apoptotic death in T leukemic cells Cell Res 2009 11 358 369 19048023 10.1038/cr.2008.314

[B18] Attia RR Gardner LA Mahrous E Taxman DJ Legros L Rowe S Ting JP Geller A Kotb M Selective targeting of leukemic cell growth in vivo and in vitro using a gene silencing approach to diminish S-adenosylmethionine synthesis J Biol Chem 2008 11 30788 30795 18753136 10.1074/jbc.M804159200 PMC2576526

[B19] Yang H Zheng Y Li TW Peng H Fernandez Ramos D Martínez-Chantar ML Rojas AL Mato JM Lu SC Methionine adenosyltransferase 2B, HuR and sirtuin 1 crosstalk impacts on Resveratrol’s effect on apoptosis and growth in liver cancer cells J Biol Chem 2013 11 23161 23170 23814050 10.1074/jbc.M113.487157 PMC3743488

[B20] Moran-Jones K Wayman L Kennedy DD Reddel RR Sara S Snee MJ Smith R HnRNP A2 a potential ssDNA/RNA molecular adapter at the telomere Nucleic Acids Res 2005 11 486 496 15659580 10.1093/nar/gki203 PMC548348

[B21] Carpenter B MacKay C Alnabulsi A MacKay M Telfer C Melvin WT Murray GI The roles of heterogeneous nuclear ribonucleoproteins in tumour development and progression Biochim Biophys Acta 2006 11 85 100 16378690 10.1016/j.bbcan.2005.10.002

[B22] Chaudhury A Chander P Howe PH Heterogeneous nuclear ribonucleoproteins (hnRNPs) in cellular processes: Focus on hnRNP E1’s multifunctional regulatory roles RNA 2010 11 1449 1462 Review 20584894 10.1261/rna.2254110 PMC2905745

[B23] Ostareck-Lederer A Ostareck DH Precision mechanics with multifunctional tools: how hnRNP K and hnRNPs E1/E2 contribute to post-transcriptional control of gene expression in hematopoiesis Curr Protein Pept Sci 2012 11 391 400 22708489 10.2174/138920312801619484

[B24] Wang H Ye Y Pan SY Zhu GY Li YW Fong DW Yu ZL Proteomic identification of proteins involved in the anticancer activities of oridonin in HepG2 cells Phytomedicine 2011 11 163 169 20724128 10.1016/j.phymed.2010.06.011

[B25] Niikura Y Ogi H Kikuchi K Kitagawa K BUB3 that dissociates from BUB1 activates caspase-independent mitotic death (CIMD) Cell Death Differ 2010 11 1011 1024 20057499 10.1038/cdd.2009.207 PMC2866768

[B26] Robertson C Church SW Nagar HA Price J Hall PA Russell SE Properties of SEPT9 isoforms and the requirement for GTP binding J Pathol 2004 11 519 527 15095474 10.1002/path.1551

[B27] Chacko AD McDade SS Chanduloy S Church SW Kennedy R Price J Hall PA Russell SE Expression of the SEPT9_i4 isoform confers resistance to microtubule-interacting drugs Cell Oncol 2012 11 85 93 10.1007/s13402-011-0066-0 PMC12995059 22278362

[B28] Kim MS Froese CD Estey MP Trimble WS SEPT9 occupies the terminal positions in septin octamers and mediates polymerization-dependent functions in abscission J Cell Biol 2011 11 815 826 22123865 10.1083/jcb.201106131 PMC3257574

[B29] Carvou N Holic R Li M Futter C Skippen A Cockcroft S Phosphatidylinositol -and phosphatidylcholine-transfer activity of PITPbeta is essential for COPI-mediated retrograde transport from the Golgi to the endoplasmic reticulum J Cell Sci 2010 11 1262 1273 20332109 10.1242/jcs.061986 PMC2848114

[B30] Snoek GT Phosphatidylinositol transfer proteins: emerging roles in cell proliferation, cell death and survival IUBMB Life 2004 11 467 475 Review 15545226 10.1080/15216540400012152

[B31] Riman S Rizkallah R Kassardjian A Alexander KE Lüscher B Hurt MM Phosphorylation of the transcription factor YY1 by CK2α prevents cleavage by caspase 7 during apoptosis Mol Cell Biol 2012 11 797 807 22184066 10.1128/MCB.06466-11 PMC3272974

[B32] Payne KJ Dovat S Ikaros and tumor suppression in acute lymphoblastic leukemia Crit Rev Oncog 2011 11 3 12 Review 22150303 10.1615/critrevoncog.v16.i1-2.20 PMC3243972

[B33] Dovat S Song C Payne KJ Li Z Ikaros, CK2 kinase, and the road to leukemia Mol Cell Biochem 2011 11 201 207 Review 21750978 10.1007/s11010-011-0964-5 PMC3665334

[B34] Wiener R Zhang X Wang T Wolberger C The mechanism of OTUB1-mediated inhibition of ubiquitination Nature 2012 11 618 622 22367539 10.1038/nature10911 PMC3319311

[B35] Sun XX Challagundla KB Dai MS Positive regulation of p53 stability and activity by the deubiquitinating enzyme Otubain 1 EMBO J 2012 11 576 592 22124327 10.1038/emboj.2011.434 PMC3273389

[B36] Ludwig H Khayat D Giaccone G Facon T Proteasome inhibition and its clinical prospects in the treatment of hematologic and solid malignancies Cancer 2005 11 1794 1797 16178003 10.1002/cncr.21414

[B37] Davis CD Tsuji PA Milner JA Selenoproteins and cancer prevention Annu Rev Nutr 2012 11 73 95 Review 22404120 10.1146/annurev-nutr-071811-150740

[B38] Carlet M Janjetovic K Rainer J Schmidt S Panzer-Grümayer R Mann G Prelog M Meister B Ploner C Kofler R Expression, regulation and function of phosphofructo-kinase/fructose-biphosphatases (PFKFBs) in glucocorticoid-induced apoptosis of acute lymphoblastic leukemia cells BMC Cancer 2010 11 638 649 21092265 10.1186/1471-2407-10-638 PMC3002928

[B39] Kowalski W Nocon D Gamian A Kołodziej J Rakus D Association of C-terminal region of phosphoglycerate mutase with glycolytic complex regulates energy production in cancer cells J Cell Physiol 2012 11 2613 2621 22367961 10.1002/jcp.22998

[B40] Hulleman E Kazemier KM Holleman A VanderWeele DJ Rudin CM Broekhuis MJ Evans WE Pieters R Den Boer ML Inhibition of glycolysis modulates prednisolone resistance in acute lymphoblastic leukemia cells Blood 2009 11 2014 2021 18978206 10.1182/blood-2008-05-157842 PMC4081395

[B41] Footz TK Brinkman-Mills P Banting GS Maier SA Riazi MA Bridgland L Hu S Birren B Minoshima S Shimizu N Pan H Nguyen T Fang F Fu Y Ray L Wu H Shaull S Phan S Yao Z Chen F Huan A Hu P Wang Q Loh P Qi S Roe BA McDermid HE Analysis of the cat eye syndrome critical region in humans and the region of conserved synteny in mice: a search for candidate genes at or near the human chromosome 22 pericentromere Genome Res 2001 11 1053 1070 11381032 10.1101/gr.154901 PMC311098

[B42] Michaud J Simpson KM Escher R Buchet-Poyau K Beissbarth T Carmichael C Ritchie ME Schütz F Cannon P Liu M Shen X Ito Y Raskind WH Horwitz MS Osato M Turner DR Speed TP Kavallaris M Smyth GK Scott HS Integrative analysis of RUNX1 downstream pathways and target genes BMC Genomics 2008 11 363 380 18671852 10.1186/1471-2164-9-363 PMC2529319

[B43] Jiang N Koh GS Lim JY Kham SK Ariffin H Chew FT Yeoh AE BIM is a prognostic biomarker for early prednisolone response in pediatric acute lymphoblastic leukemia Exp Hematol 2011 11 321 329 21130142 10.1016/j.exphem.2010.11.009

[B44] Trembley JH Chen Z Unger G Slaton J Kren BT Van Waes C Ahmed K Emergence of protein kinase CK2 as a key target in cancer therapy Biofactors 2010 11 187 195 20533398 10.1002/biof.96 PMC2916697

[B45] Polak R Buitenhuis M The PI3K/PKB signaling module as key regulator of hematopoiesis: implications for therapeutic strategies in leukemia Blood 2012 11 911 923 22065598 10.1182/blood-2011-07-366203

[B46] Ernst A Avvakumov G Tong J Fan Y Zhao Y Alberts P Persaud A Walker JR Neculai AM Neculai D Vorobyov A Garg P Beatty L Chan PK Juang YC Landry MC Yeh C Zeqiraj E Karamboulas K Allali-Hassani A Vedadi M Tyers M Moffat J Sicheri F Pelletier L Durocher D Raught B Rotin D Yang J Moran MF A strategy for modulation of enzymes in the ubiquitin system Science 2013 11 590 595 23287719 10.1126/science.1230161 PMC3815447

[B47] Schneider P Costa O Legrand E Bigot D Lecleire S Grassi V Vannier JP Vasse M In vitro secretion of matrix metalloprotease 9 is a prognostic marker in childhood acute lymphoblastic leukemia Leuk Res 2010 11 24 31 19748669 10.1016/j.leukres.2009.07.039

[B48] Gandemer V Chevret S Petit A Vermylen C Leblanc T Michel G Schmitt C Lejars O Schneider P Demeocq F Bader-Meunier B Bernaudin F Perel Y Auclerc MF Cayuela JM Leverger G Baruchel A FRALLE Group Excellent prognosis of late relapses of ETV6/RUNX1-positive childhood acute lymphoblastic leukemia: lessons from the FRALLE 93 protocol Haematologica 2012 11 1743 1750 22580999 10.3324/haematol.2011.059584 PMC3487450

[B49] Van Dongen JJ Macintyre EA Gabert JA Delabesse E Rossi V Saglio G Gottardi E Rambaldi A Dotti G Griesinger F Parreira A Gameiro P Diáz MG Malec M Langerak AW San Miguel JF Biondi A Standardized RT-PCR analysis of fusion gene transcripts from chromosome aberrations in acute leukemia for detection of minimal residual disease. Report of the BIOMED-1 Concerted Action: investigation of minimal residual disease in acute leukemia Leukemia 1999 11 1901 1928 10602411 10.1038/sj.leu.2401592

[B50] Schneider P Vasse M Corbière C Legrand E Marie-Cardine A Boquet C Vannier JP Endostatin variations in childhood acute lymphoblastic leukaemia-comparison with basic fibroblast growth factor and vascular endothelial growth factor Leuk Res 2007 11 629 638 17011029 10.1016/j.leukres.2006.08.023

[B51] Tnani H López I Jouenne T Vicienta CM Protein composition analysis of oil bodies from maize embryos during germination J Plant Physiol 2011 11 510 513 21247659 10.1016/j.jplph.2010.08.020

[B52] Magdeldin S Enany S Yoshida Y Xu B Zhang Y Zureena Z Lokamani I Yaoita E Yamamoto T Basics and recent advances of two dimensional-polyacrylamide gel electrophoresis Clin Proteomics 2014 11 16 26 24735559 10.1186/1559-0275-11-16 PMC3996944

