# Supplementary material for: Proteomic profile of pre - B2 lymphoblasts from children with acute lymphoblastic leukemia (ALL) in relation with the translocation (12; 21)
Source: Clin Proteomics. 2014 Aug 1;11(1):31. doi: 10.1186/1559-0275-11-31 (PMC4128613; doi:10.1186/1559-0275-11-31)
Supplement: Additional file 1: Table S1 — Classification of differentially expressed proteins in pre-B2 lymphoblastic cells from children with ALL. 2-DE Batches are located Figure 1b. R1, R2 and R3 (In protein names column) are the Mascot rank basis on the whole ionic scores (IS) detected (Here only IS, listed in Additional file 1: Figure S3, upper or equal to 48 are taken in consideration); Power analysis was performed independently for each spot, taking into consideration the sample size and variance expression value (Progenesis SameSpots V4). Figure S1. Normalized volume of silver stained spots from two dimensional gels: A- Average normalized volume realized from 9 patients which were chosen randomly. Gels were performed in triplicate (bars: SD, n=3). Spot A to spot L were chosen randomly to valid the method. B- Normalized volume realized from 13 patients included in final statistical analysis (referred in the main manuscript Figure 3), focusing on the 11 first spots discussed in the main document. Values of three 2D maps for each ALL patients with t(12;21) (Ba), or ALL patients without this translocation (Bb). Figure S2. Spot localization on 2-DE gels: Only spots submitted with success to the student t test values (p>0.01 and p≤ 0.05) are numbered. Numbers correspond to protein spots listed in Table 2. Figure S3. Mascot research results for the first eleven spot proteins ordered by statistic rank (Progenesis SameSpotsV4 software –see Additional file 1: Table S1). For each identified trypsic peptide, individual ions scores up to 48 indicate identity or extensive homology (p<0.05). Matched peptides are shown in bold red. [file 1559-0275-11-31-S1.pdf]

Odile Costa et al, Clinical Protemonics

Additional file 1

Additional table 1s. **Classification of differentially expressed proteins in pre-B2 lymphoblastic cells from children with ALL.** 2-DE Batches are located fig 1b. R1, R2 and R3 (In protein names column) are the Mascot rank basis on the whole ionic scores (IS) detected (Here only IS, listed in figure 3s, upper or equal to 48 are taken in consideration); Power analysis was performed independently for each spot, taking into consideration the sample size and variance expression value (Progenesis SameSpots V4).

Spot ordered by Statistic Rank (2DE batch)	Protein names [Homo sapiens]	NCBI and Uniprot ID numbers	M W (kDa x 10^3)	Isofocusing Point	Sequence coverage	Number of identified Ion Scores (IS)-total score (number of individual IS)	Fold	Group with the Upper Average Volume	Student t Test	Power (after q value)	Major field of activity
1 (2768)	Calponin-2 (CNN2)	gi/49456619 - Q99439	33,70	6.95	32%	303 (5 IS)	1.9	Pre B2, t(12;21)	p≤ 0.001	0.998	Cell cycle regulation
2 (3185)	Cysteine desulfurase, mitochondrial isoform a (CDSa)	gi/32307132 - Q9Y697-1	50,10	8.54	24%	248 (4 IS)	2.2	Pre B2 with other karyotype abnormalities	p≤ 0.001	0.985	Metabolism
3 (4083)	Phosphatidylinositol transfer protein beta isoform (PITPB)	gi/6912594 - P48739	31,50	6.41	45%	242 (4 IS)	2	Pre B2, t(12;21)	p≤ 0.001	0.983	Apoptosis
4 (2457)	R1: hnRNP-E1	gi/460771 - Q15365	37,50	6.66	56%	273 (5 IS)	1.4	Pre B2 with other karyotype abnormalities	p≤ 0.005	0.976	Cell cycle regulation
	R2 : Mitotic checkpoint protein BUB3 isoform a (BUB3a)	*gi/4757880 -* O43684	*37,10*	*6.36*	*35%*	170 (3 IS)					Cell cycle regulation
5 (4069)	Chain A, Pyruvate Dehydrogenase S264e Variant (PDH)	gi/149242791- P13804 -1	40,70	6.48	35%	541 (7 IS)	1.5	Pre B2 with other karyotype abnormalities	p≤ 0.005	0.955	Metabolism
6 (2090)	Methionine adenosyltransferase 2 subunit beta isoform 1 (MAT2B)	gi/11034825 - Q9NZL9	37,50	6.9	44%	388 (6 IS)	1.5	Pre B2, t(12;21)	p≤ 0.005	0.915	cell cycle regulation
7 (2800)	Proteasome subunit beta type-2 isoform 1 (PSMB2)	gi/4506195 - B7Z478	22,80	6.51	39%	183 (3 IS)	1.7	Pre B2, t(12;21)	p≤ 0.01	0.878	Apoptosis
8 (4081)	R1 : Cat eye syndrome critical region protein 5 isoform 2 precursor (*CECR5*)	gi/14861834 - Q9BXW7	46,30	8.38	39%	375 (6 IS)	1.3	Pre B2 with other karyotype abnormalities	p≤ 0.01	0.867	Posttranslational modification
	R2: Mitotic checkpoint protein BUB3 isoform a(BUB3a)	*gi/4757880 -* O43684	*37,10*	*6.36*	*33%*	265 (5 IS)					Cell cycle regulation
9 (3121)	R1: casein kinase II subunit alpha isoform a (CK2a)	gi/4503095 - P68400	45,10	7.23	25%	195 (3 IS)	1.5	Pre B2 with other karyotype abnormalities	p≤ 0.01	0.864	Apoptosis
	R2: MLL septin-like fusion protein MSF-B (SEPT9_i3)	*gi/6683815 -* Q9UHD8-3	*47,40*	*6.21*	*32%*	169 (3 IS)					Cell cycle regulation
10 (2056)	Heterogeneous nuclear ribonucleoproteins A2/B1 isoform A2	gi/4504447 - P22626-2	41,76	9,32	*48%*	242 (4 IS)	2	Pre B2, t(12;21)	p≤ 0.01	0.854	Cell cycle regulation
11 (3120)	R1: Chain A, Isovaleryl-Coa Dehydrogenase (IVAD)	gi/3212539 - P26440	43,00	6.9	42%	272 (3 IS)	1.5	Pre B2 with other karyotype abnormalities	p≤ 0.01	0.835	Metabolism
	R2: Fructose bisphosphate aldolase	gi/312137 - P05062	39,40	6.41	29%	338 (5 IS)					Metabolism
	R3: Proteasome subunit p42 (PSMB6)	gi/1526426 - P62333	44,10	7.1	33%	247 (5 IS)					Apoptosis
12(2656)	HSPC263/Deubiquitinating enzyme (OTUB1)	gi/6841176 - Q96FW1	31,67	4,9	47%	264(4 IS)	1,8	Pre B2, t[12;21]	p≤ 0,05	0,787	Ubiquitin-proteasome system
13(1455)	NI						1,9	Pre B2 with other karyotype anomalies	p≤ 0,05	0,768	NI
14 (3247)	R1 : Chain A, Human Muscle Fructose 1,6-Bisphosphate Aldolase	gi/4557976 - P04075	39,27	8,39	66%	670 (11 IS)	1,4	Pre B2 with other karyotype anomalies	p≤ 0,05	0,727	Metabolism
	R2 : NAD+-specific isocitrate dehydrogenase beta precursor [Homo sapiens] (IDHB3)	g/2737886 - O43837	42,19	8,64	43%	161 (3 IS)					Metabolism
15 (4045)	Exosome complex component RRP4	gi/19923403 - Q13868	32,77	7,06	54%	279 (4 IS)	1,3	Pre B2 with other karyotype anomalies	p≤ 0,05	0,722	Degradation and processing of cellular RNA
16 (1149)	NI						1,6	Pre B2, t[12;21]	p≤ 0,05	0,722	NI
17 (3519)	Isocitrate dehydrogenase alpha (IDH)	gi/5031777 - P50213-1	39,57	6,47	47%	833 (9 IS)	1,4	Pre B2 with other karyotype anomalies	p≤ 0,05	0,692	Metabolism
18 (3077)	Exosome complex component RRP4	gi/19923403 - Q13868	32,77	7,06	45%	184 (3 IS)	1,4	Pre B2 with other karyotype anomalies	p≤ 0,05	0,685	Degradation and processing of cellular RNA
19 (2808)	Chain A, Three-Dimensional Structure Of Human Electron Transfer Flavoprotein To 2.1 A Resolution	gi/2781202 - P13804-1	33,08	9,95	28%	152 (4 IS)	1,4	Pre B2 with other karyotype anomalies	p≤ 0,05	0,684	Metabolism - Electron transfert
20 (2725)	Prohibitin [Homo sapiens] (PHB)	gi/46360168 - P35232	29,80	5,57	76%	775 (10 IS)	1,2	Pre B2 with other karyotype anomalies	p≤ 0,05	0,664	Cell cycle regulation
21 (3000)	Chain A human electron transfer flavoprotein	gi/2781202 - P13804-1	33,08	6,95	53%	247 (4 IS)	1,4	Pre B2 with other karyotype anomalies	p≤ 0,05	0,655	Metabolism - Electron transfert
22 (1439)	NI						1,9	Pre B2 with other karyotype anomalies	p≤ 0,05	0,649	
23 (3437)	Tapasin ER57/ER60 (58 kDa glucose-regulated protein)	gi/220702506 - P30120	54,20	5,61	50%	576 (10 IS)	1,3	Pre B2 with other karyotype anomalies	p≤ 0,05	0,648	Metabolism (Disulfure isomerase)
24 (3999)	Chain A, Structure Of Human Glutamate Dehydrogenase-Apo Form	gi/20151189 - P00367	55,97	6,71	59%	436 (7 IS)	1,3	Pre B2 with other karyotype anomalies	p≤ 0,05	0,601	Metabolism
25 (3486)	Elongation factor Tu	gi/704416 - P49411	49,51	7,7	70%	899 (14 IS)	1,2	Pre B2 with other karyotype anomalies	p≤ 0,05		Protein biosynthesis
26 (3076)	Chain A, Three-Dimensional Structure Of Human Electron Transfer Flavoprotein	gi/2781202 - P13804 -1	33,08	6,95	25%	205 (3 IS)	1,2	Pre B2 with other karyotype anomalies	p≤ 0,05	0,537	Metabolism - Electron transfert
	Exosome complex component RRP4	gi/19923403 - Q13868	32,77	7,06	56%	454 (4 IS)		Pre B2 with other karyotype anomalies			Degradation and processing of cellular RNA

Additional figure 1s **– Normalized volume of silver stained spots from two dimensional gels: A-** Average normalized volume realized from 9 patients which were chosen randomly. Gels were performed in triplicate (bars: SD, n=3). Spot A to spot L were chosen randomly to valid the method. **B-** Normalized volume realized from 13 patients included in final statistical analysis (*referred in the main manuscript figure 3*), focusing on the 11 first spots discussed in the main document. Values of three 2D maps for each ALL patients with t(12;21) (Ba), or ALL patients without this translocation (Bb).

Figure 1s-A-

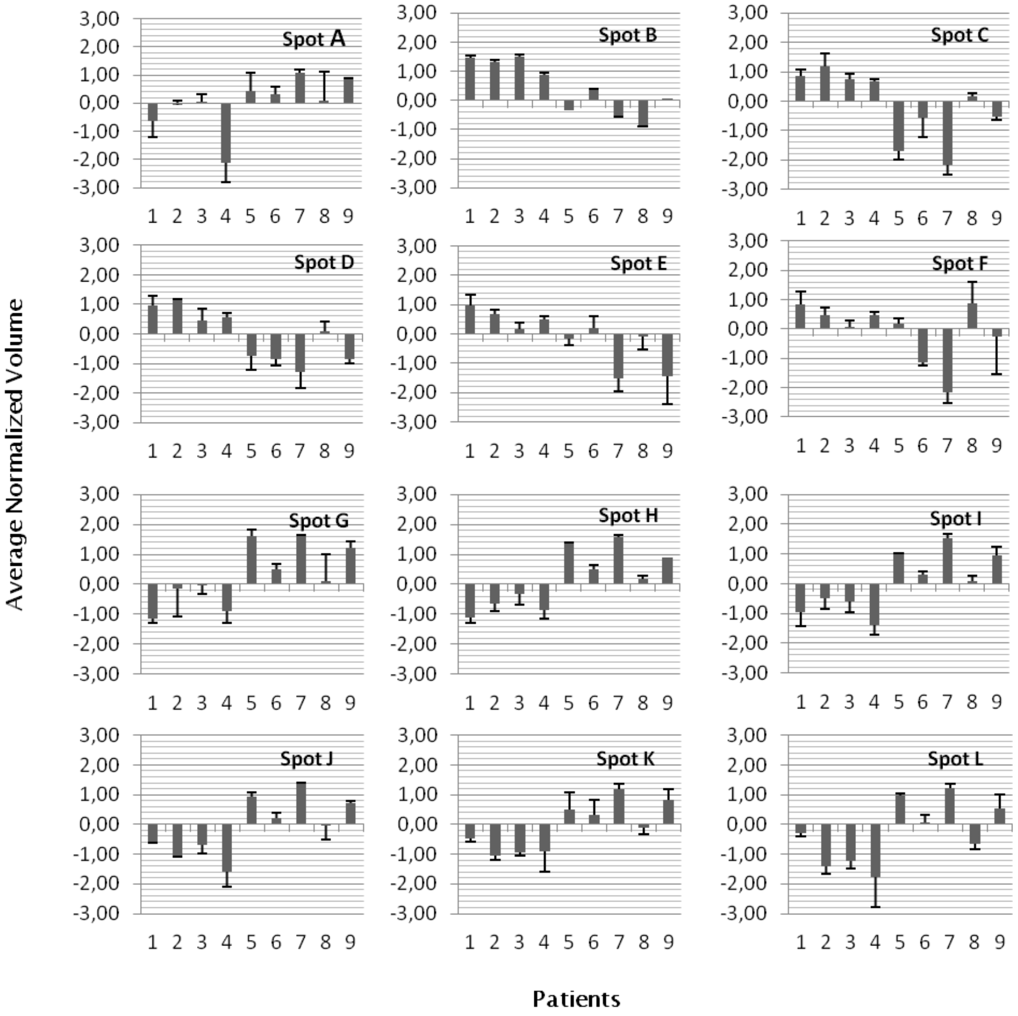

Figure 1s-B-

Figure 1s-Ba

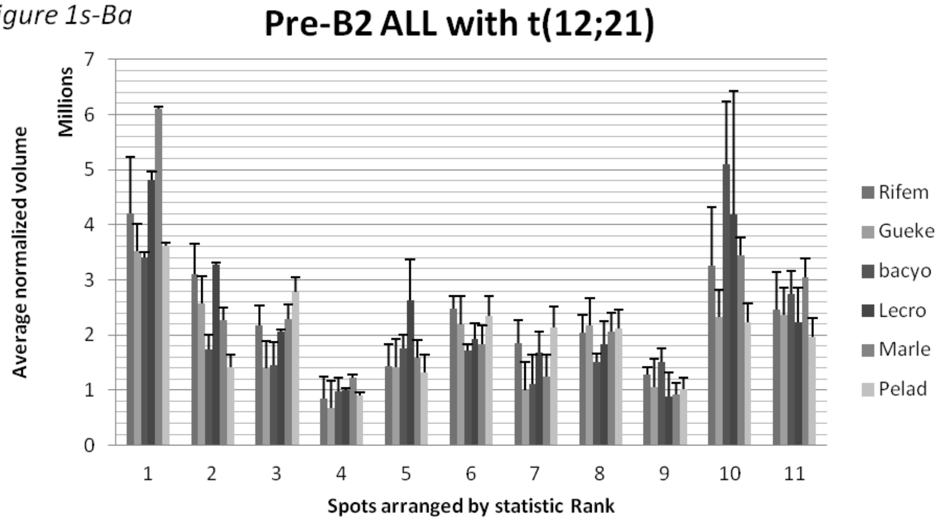

Figure 1s-Bb

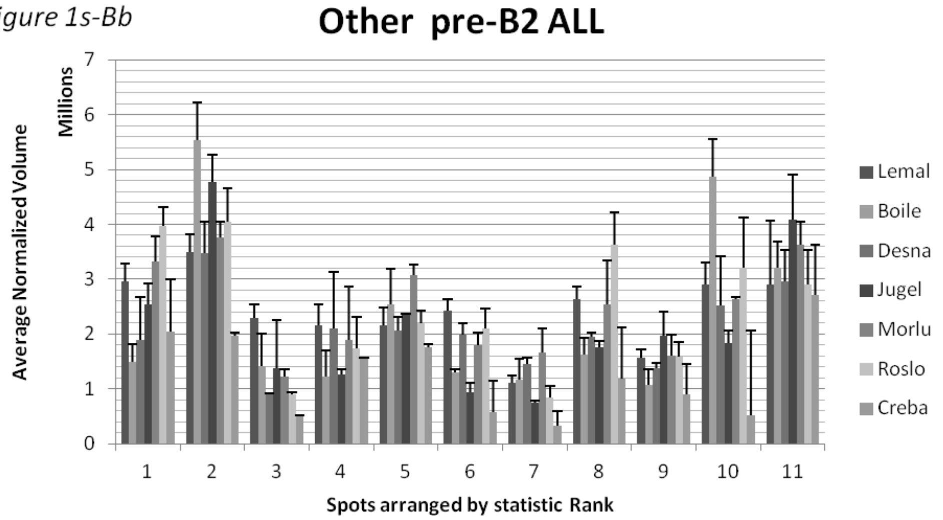

***Additional figure 2s* - Spot localization on 2-DE gels**: Only spots submitted with success to the student t test values (p>0.01 and p≤ 0.05) are numbered. Numbers correspond to protein spots listed in table 2.

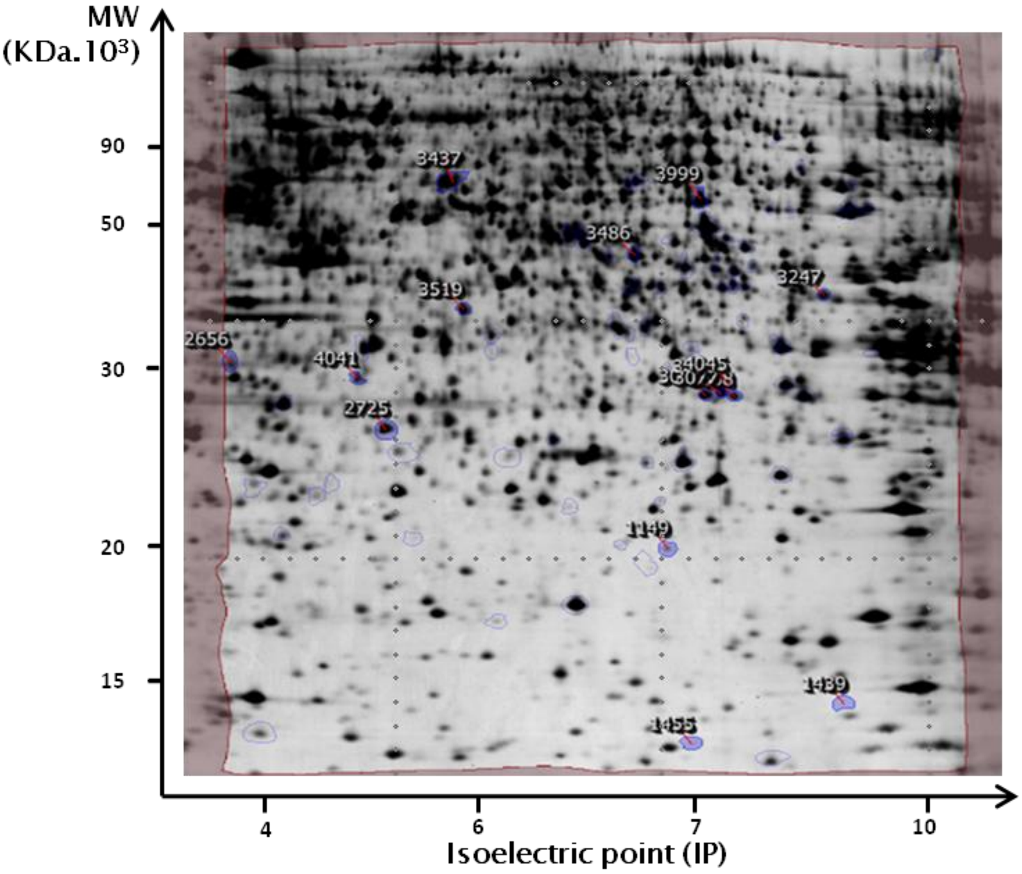

***Additional figure 3s:* Mascot research results for the first eleven spot proteins ordered by statistic rank (*Progenesis SameSpotsV4 software –see Additional table 1s*).** For each identified trypsic peptide, individual ions scores up to 48 indicate identity or extensive homology (p<0.05). Matched peptides are shown in bold red.

Calponin -2 (2-DE batch: 2768; rank 1) :

- *List of tryptic peptides recovered:*

Query	Observed	Mr(expt)	Mr(calc)	Delta	Miss	Score	Expect	Rank	Peptide
160	388.9800	775.9454	775.3864	0.5590	0	22	25	2	R.HIYDTK.L
248	479.6300	957.2454	956.3909	0.8545	0	9	4.2e+02	3	R.NFDDATMK.A + Oxidation (M)
300	536.8500	1071.6854	1071.5924	0.0930	0	55	0.011	1	**K.GLQSGVDIGVK.Y**
310	554.9900	1107.9654	1106.5608	1.4047	0	55	0.012	1	**K.GPSYGLSAEVK.N**
368	624.7900	1247.5654	1247.6146	-0.0491	1	46	0.085	1	**K.YDPQKEAELR.T**
374	641.7200	1281.4254	1280.6105	0.8150	0	52	0.019	1	**K.DGTILCTLMNK.L + Carbamidomethyl (C); Oxidation (M)**
407	676.3800	1350.7454	1351.5860	-0.8406	0	(10)	3.3e+02	7	K.CSSQVGMTAPGTR.R + Carboxymethyl (C)
415	684.2800	1366.5454	1366.5970	-0.0515	0	34	1.5	1	**K.CSSQVGMTAPGTR.R + Carbamidomethyl (C); Oxidation (M)**
424	703.2900	1404.5654	1404.5762	-0.0108	0	78	5.3e-05	1	**K.CASQSGMTAYGTR.R + Carbamidomethyl (C); Oxidation (M)**
439	746.4500	1490.8854	1491.7174	-0.8319	0	63	0.0016	1	**K.AGQCVIGLQMGTNK.C + Carbamidomethyl (C); Oxidation (M)**
454	790.9300	1579.8454	1578.8109	1.0345	1	34	1.1	1	**K.GLKDGTILCTLMNK.L + Carbamidomethyl (C); Oxidation (M)**

- *Protein sequence with matched peptides in bold red:*

1 MSSTQFNK**GP SYGLSAEVK**N RLLSK**YDPQK EAELR**TWIEG LTGLSIGPDF
51 QK**GLKDGTIL CTLMNK**LQLG SVPKINRSMQ NWHQLENLSN FIKAMVSYGM
101 NPVDLFEAND LFESGNMTQV QVSLLALAGK AKTK**GLQSGV DIGVK**YSEKQ
151 ER**NFDDATMK AGQCVIGLQM GTNKCASQSG MTAYGTR**RHL YDPKNHILPP
201 MDHSTISLQM GTNK**CSSQVG MTAPGTR**R**HI YDTK**LGTDKC DNSSMSLQMG
251 YTQGANQSGQ VFGLGRQIYD PKYCPQGTVA DGAPSGTGDC PDPGEVPEYP
301 PYYQEEAGY

- **Example of MS/MS Fragmentation :** MS/MS Fragmentation of **CASQSGMTAYGTR** peptide [from 175 to 187]:

Match to Query 424: 1404.565448 from(703.290000,2+) intensity(5016155.0000)
Title: Cmpd 106, +MSn(703.7), 3.0 min
Data file F:\Labos IRIB\Odile Costa\PC10Avril2012\120416_COQUET\OC_32.mgf

Click mouse within plot area to zoom in by factor of two about that point
Or, Plot from 200 to 1200 Da Full range

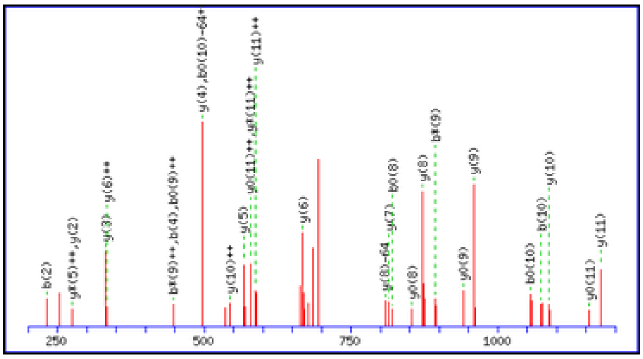

Monoisotopic mass of neutral peptide Mr(calc): 1404.5762
Variable modifications:
C1 : Carbamidomethyl (C)
M7 : Oxidation (M), with neutral losses 0.0000(shown in table), 63.9983
Ions Score: 78 Expect: 5.3e-05
Matches (**Bold Red**): 30/204 fragment ions using 40 most intense peaks

#	b	b^{++}	b^{*}	b^{*++}	b^{0}	b^{0++}	Seq.	y	y^{++}	y^{*}	y^{*++}	y^{0}	y^{0++}	#
1	161.0379	81.0226					C							13
2	**232.0750**	116.5412					A	1245.5528	623.2801	1228.5263	614.7668	1227.5423	614.2748	12
3	319.1071	160.0572			301.0965	151.0519	S	**1174.5157**	**587.7615**	1157.4892	**579.2482**	**1156.5052**	**578.7562**	11
4	**447.1656**	224.0865	430.1391	215.5732	429.1551	215.0812	Q	**1087.4837**	**544.2455**	1070.4571	535.7322	1069.4731	535.2402	10
5	534.1977	267.6025	517.1711	259.0892	516.1871	258.5972	S	**959.4251**	480.2162	942.3986	471.7029	**941.4145**	471.2109	9
6	591.2191	296.1132	574.1926	287.5999	573.2086	287.1079	G	**872.3931**	436.7002	855.3665	428.1869	**854.3825**	427.6949	8
7	738.2545	369.6309	721.2280	361.1176	720.2440	360.6256	M	**815.3716**	408.1894	798.3451	399.6762	797.3611	399.1842	7
8	839.3022	420.1548	822.2757	411.6415	**821.2917**	411.1495	T	**668.3362**	**334.6717**	651.3097	326.1585	650.3257	325.6665	6
9	910.3393	455.6733	**893.3128**	**447.1600**	892.3288	**446.6680**	A	**567.2885**	284.1479	550.2620	**275.6346**	549.2780	275.1426	5
10	**1073.4027**	537.2050	1056.3761	528.6917	**1055.3921**	528.1997	Y	**496.2514**	248.6293	479.2249	240.1161	478.2409	239.6241	4
11	1130.4241	565.7157	1113.3976	557.2024	1112.4136	556.7104	G	**333.1881**	167.0977	316.1615	158.5844	315.1775	158.0924	3
12	1231.4718	616.2395	1214.4453	607.7263	1213.4612	607.2343	T	**276.1666**	138.5870	259.1401	130.0737	258.1561	129.5817	2
13							R	175.1190	88.0631	158.0924	79.5498			1

Cysteine desulfurase (2-DE batch: 3185; rank 2)

- *List of tryptic peptides recovered:*

Query	Observed	Mr(expt)	Mr(calc)	Delta	Miss	Score	Expect	Rank	Peptide
181	425.1400	848.2654	847.4916	0.7739	0	27	6.7	1	K.GVGAIYIR.R
214	454.8900	907.7654	908.3844	-0.6190	0	22	22	1	K.CVLDSCR.S + 2 Carbamidomethyl (C)
273	530.7100	1059.4054	1059.5270	-0.1216	0	30	3.2	2	K.IPLDVNDMK.I + Oxidation (M)
171	403.4200	1207.2382	1206.6357	0.6025	0	28	5.8	1	K.HLITTQTEHK.C
347	615.8300	1229.6454	1229.6000	0.0454	0	64	0.0014	1	R.VEALQSGGGQER.G
354	627.8300	1253.6454	1253.6728	-0.0273	0	(42)	0.2	1	R.QQVASLIGADPR.E
355	627.8800	1253.7454	1253.6728	0.0727	0	51	0.023	1	R.QQVASLIGADPR.E
363	644.2300	1286.4454	1286.6177	-0.1722	0	33	1.4	1	K.SLPDVVMNGDPK.H + Oxidation (M)
416	742.8600	1483.7054	1483.7267	-0.0212	0	62	0.002	1	R.AIGTDEDLAHSSIR.F
253	511.9100	1532.7082	1533.7940	-1.0858	1	33	1.8	1	R.KVYFHTDAAQAVGK.I
447	795.3500	1588.6854	1588.7144	-0.0290	0	71	0.00024	1	R.FTTEEEVDYTVEK.C

- *Protein sequence with matched peptides in bold red:*

```
  1 MLLRAAWRRA AVAVTAAPGP KPAAPTRGLR LRVGDRAPQS AVPADTAAAP
 51 EVGPVLRPLY MDVQATTPLD PRVLDAMLPY LINYYGNPHS RTHAYGWESE
101 AAMERARQQV ASLIGADPRE IIFTSGATES NNIAIKGVAR FYRSRKKHLI
151 TTQTEHKCVL DSCRSLEAEG FQVTYLPVQK SGIIDLKELE AAIQPDTSLV
201 SVMTVNNEIG VKQPIAEIGR ICSSRKVYFH TDAAQAVGKI PLDVNDMKID
251 LMSISGHKIY GPKGVGAIYI RRRPRVRVEA LQSGGGQERG MRSGTVPTPL
301 VVGLGAACEV AQQEMEYDHK RISKLSERLI QNIMKSLPDV VMNGDPKHHY
351 PGCINLSFAY VEGESLLMAL KDVALSSGSA CTSASLEPSY VLRAIGTDED
401 LAHSSIRFGI GRFTTEEEVD YTVEKCIQHV KRLREMSPLW EMVQDGIDLK
451 SIKWTQH
```

Phosphatidylinositol transfer protein beta (2-DE batch: 4083 ; rank 3):

- *List of tryptic peptides recovered:*

Query	Observed	Mr(expt)	Mr(calc)	Delta	Miss	Score	Expect	Rank	Peptide
114	344.5900	687.1654	687.4068	-0.2414	0	21	27	1	K.VPAFVR.M
162	392.5300	783.0454	783.3803	-0.3349	0	20	34	1	K.DDFFIK.I
189	439.8100	877.6054	876.4705	1.1349	0	39	0.51	1	K.VENFIQK.Q
223	468.1600	934.3054	933.4821	0.8234	0	37	0.68	1	R.IFTNFHR.Q
238	518.5000	1034.9854	1035.4873	-0.5018	0	17	66	1	R.SQVEPADYK.A
272	557.9700	1113.9254	1113.5376	0.3878	0	30	3.4	1	R.TIVTNEYMK.D + Oxidation (M)
333	651.8300	1301.6454	1301.6463	-0.0009	0	71	0.00029	1	K.NETGGGEGIEVLK.N
341	660.3200	1318.6254	1318.6405	-0.0150	0	52	0.024	1	K.ADEDPALFQSVK.T
380	690.9000	1379.7854	1379.7409	0.0446	0	65	0.00094	1	K.TVEIVHIDIADR.S
407	737.4400	1472.8654	1472.7333	0.1321	0	(18)	41	1	R.MIAPEGSLVFHEK.A + Oxidation (M)
230	492.3200	1473.9382	1472.7333	1.2048	0	31	2.7	1	R.MIAPEGSLVFHEK.A + Oxidation (M)
450	836.4300	1670.8454	1670.7127	0.1327	1	27	5.6	1	R.MEDETQKELETMR.K + 2 Oxidation (M)
468	900.4100	1798.8054	1798.7324	0.0730	0	54	0.01	1	K.ELANSPDCPQMCAYK.L + 2 Carbamidomethyl (C); Oxidation (M)
323	627.3600	1879.0582	1878.9073	0.1508	1	18	47	3	R.TIVTNEYMKDDFFIK.I + Oxidation (M)

- *Protein sequence with matched peptides in bold red:*

  1 MVLIKEFRVV LPCSVQEYQV GQLYSVAEAS K**NETGGGEGI EVLK**NEPYEK
 51 DGEKGQYTHK IYHLKSK**VPA FVRMIAPEGS LVFHEK**AWNA YPYCR**TIVTN**
101 **EYMKDDFFIK** IETWHKPDLG TLENVHGLDP NTWK**TVEIVH IDIADRSQVE**
151 **PADYKADEDP ALFQSVK**TKR GPLGPNWKKE **LANSPDCPQM CAYK**LVTIKF
201 KWWGLQSK**VE NFIQK**QEKR**I FTNFHR**QLFC WIDKWIDLTM EDIRR**MEDET**
251 **QKELETMR**KR GSVRGTSAAD V

Heterogeneous nuclear ribonucleoprotein-E1 (2-DE batch: 2457; rank 4-1):

- *List of tryptic peptides recovered:*

Query	Observed	Mr(expt)	Mr(calc)	Delta	Miss	Score	Expect	Rank	Peptide
66	329.6600	657.3054	657.4173	-0.1119	1	26	9.7	1	K.IKEIR.E
72	350.6000	699.1854	699.4028	-0.2173	0	29	4.6	2	K.LNQVAR.Q
92	402.3900	802.7654	801.4596	1.3058	0	21	31	1	K.EVGSIIGK.K
119	439.8600	877.7054	877.4327	0.2727	0	39	0.47	1	R.QMSGAQIK.I + Oxidation (M)
140	459.8100	917.6054	916.4726	1.1328	1	29	3.9	1	R.IREESGAR.I
163	508.1400	1014.2654	1013.5254	0.7401	0	33	1.6	1	R.QGANINEIR.Q
190	543.9900	1085.9654	1085.5465	0.4189	0	56	0.0088	1	K.IANPVEGSSGR.Q
191	544.0500	1086.0854	1085.5465	0.5389	0	(15)	1.3e+02	1	K.IANPVEGSSGR.Q
242	644.7800	1287.5454	1287.5877	-0.0423	0	50	0.035	1	R.INISEGNCPER.I + Carbamidomethyl (C)
265	694.9100	1387.8054	1387.8075	-0.0020	0	49	0.042	1	R.IITLTGPTNAIFK.A
281	721.9300	1441.8454	1441.7963	0.0492	0	39	0.38	1	R.LVVPATQCGSLIGK.G + Carbamidomethyl (C)
356	836.9500	1671.8854	1671.8866	-0.0011	0	42	0.18	1	R.AITIAGVPQSVTECVK.Q + Carbamidomethyl (C)
440	980.2900	1958.5654	1958.9918	-0.4263	0	(21)	21	1	K.QICLVMLETLSQSPQGR.V + Carbamidomethyl (C)
442	988.4700	1974.9254	1974.9867	-0.0612	0	49	0.032	1	K.QICLVMLETLSQSPQGR.V + Carbamidomethyl (C); Oxidation (M)
458	1053.6000	2105.1854	2104.9695	0.2159	0	(54)	0.011	1	R.ESTGAQVQVAGDMLPNSTER.A + Oxidation (M)
270	702.7800	2105.3182	2104.9695	0.3487	0	69	0.00035	1	R.ESTGAQVQVAGDMLPNSTER.A + Oxidation (M)
390	874.7800	2621.3182	2621.1639	0.1543	0	33	1.6	1	R.QQSHFAMMHGGTGFAGIDSSSPEVK.G + Oxidation (M)
394	880.4000	2638.1782	2637.1588	1.0194	0	(24)	12	1	R.QQSHFAMMHGGTGFAGIDSSSPEVK.G + 2 Oxidation (M)
478	1137.9400	3410.7982	3409.6857	1.1125	1	15	74	1	K.AFAMIIDKLEEDINSSMTNSTAASRPPVTLR.L + 2 Oxidation (M)

- *Protein sequence with matched peptides in bold red:*

  1 MDAGVTESGL NVTLTIRLLM HGK**EVGSIIG K**KGESVKR**IR EESGARINIS**
 51 **EGNCPERIIT LTGPTNAIFK AFAMIIDKLE EDINSSMTNS TAASRPPVTL**
101 **RLVVPATQCG SLIGK**GGCKI **KEIRESTGAQ VQVAGDMLPN STERAITIAG**
151 **VPQSVTECVK QICLVMLETL SQSPQGR**VMT IPYQPMPASS PVICAGGQDR
201 CSDAVGYPHA THDLEGPPLD AYSIQGQHTI SPLDLAK**LNQ VARQQSHFAM**
251 **MHGGTGFAGI DSSSPEVK**GY WASLDASTQT THELTIPNNL IGCIIGR**QGA**
301 **NINEIRQMSG AQIKIANPVE GSSGR**QVTII GSAASISLAQ YLINARLSSE
351 KGMGCS

Mitotic checkpoint protein BUB3 a (2-DE batch: 2457; rank 4-2):

- *List of tryptic peptides recovered:*

Query	Observed	Mr(expt)	Mr(calc)	Delta	Miss	Score	Expect	Rank	Peptide
99	393.9100	785.8054	785.4759	0.3295	0	24	12	1	R.LIVGTAGR.R
121	431.2900	860.5654	859.4123	1.1532	0	42	0.23	1	R.LCQFHR.Y + Carbamidomethyl (C)
171	506.5900	1011.1654	1010.4604	0.7051	0	49	0.042	1	R.NMGYVQQR.R + Oxidation (M)
205	549.0100	1096.0054	1095.5560	0.4494	0	67	0.00062	1	K.VYTLSVSGDR.L
92	372.5800	1114.7182	1115.5822	-0.8641	0	(25)	12	1	R.QVTDAETKPK.S
212	558.9900	1115.9654	1115.5822	0.3832	0	26	7.7	1	R.QVTDAETKPK.S
231	591.3400	1180.6654	1180.5546	0.1108	0	24	11	1	R.LYDVPANSMR.L + Oxidation (M)
245	604.8900	1207.7654	1207.6197	0.1458	0	49	0.039	1	K.QGYVLSSIEGR.V
303	718.9000	1435.7854	1435.6402	0.1453	0	52	0.022	1	R.TPCNAGTFSQPEK.V + Carbamidomethyl (C)
352	787.4500	1572.8854	1572.8035	0.0819	0	48	0.05	1	R.VAVEYLDPSPEVQK.K
395	851.5700	1701.1254	1700.8985	0.2270	1	(20)	28	1	R.VAVEYLDPSPEVQKK.K
219	568.0900	1701.2482	1700.8985	0.3497	1	47	0.081	1	R.VAVEYLDPSPEVQKK.K
332	759.2400	2274.6982	2275.0651	-0.3670	0	35	0.95	1	K.MHDLNTDQENLVGTHDAPIR.C
337	764.6800	2291.0182	2291.0601	-0.0419	0	(29)	3.4	1	K.MHDLNTDQENLVGTHDAPIR.C + Oxidation (M)

- *Protein sequence with matched peptidesin bold red:*

1 MTGSNEFKLN QPPEDGISSV KFSPNTSQFL LVSSWDTSVR **LYDVPANSMR**

51 LKYQHTGAVL DCAFYDPTHA WSGGLDHQLK **MHDLNTDQEN LVGTHDAPIR**

101 CVEYCPEVNV MVTGSWDQTV KLWDPR**TPCN AGTFSQPEKV YTLSVSGDRL**

151 **IVGTAGR**RVL VWDLR**NMGYV QQR**RESSLKY QTRCIRAFPN K**QGYVLSSIE**

201 **GRVAVEYLDP SPEVQKK**KYA FKCHRLKENN IEQIYPVNAI SFHNIHNTFA

251 TGGSDGFVNI WDPFNKKR**LC QFHR**YPTSIA SLAFSNDGTT LAIASSYMYE

301 MDDTEHPEDG IFIR**QVTDAE TKPK**SPCT

Chain A, Pyruvate Dehydrogenase (2-DE batch: 4069; Rank 5):

- *List of tryptic peptides recovered:*

Query	Observed	Mr(expt)	Mr(calc)	Delta	Miss	Score	Expect	Rank	Peptide
184	399.9600	797.9054	796.3571	1.5483	0	22	21	2	R.MMQTVR.R + 2 Oxidation (M)
219	437.6000	873.1854	873.4709	-0.2854	0	19	48	1	R.GDFIPGLR.V
265	466.7800	931.5454	931.5048	0.0406	0	31	2.7	1	K.SDPIMLLK.D + Oxidation (M)
267	469.1900	936.3654	935.4614	0.9041	0	6	7.7e+02	10	R.AHGFTFTR.G
278	495.6000	989.1854	988.4502	0.7353	0	46	0.092	1	R.AAASTDYYK.R
280	501.6000	1001.1854	1000.5553	0.6302	0	46	0.078	1	R.EILAELTGR.K
283	508.6400	1015.2654	1014.4440	0.8214	0	56	0.0086	1	R.YGMGTSVER.A + Oxidation (M)
336	573.4600	1144.9054	1144.5513	0.3542	1	75	0.00012	1	R.AAASTDYYKR.G
337	574.4700	1146.9254	1146.6318	0.2936	1	20	37	1	R.SKSDPIMLLK.D + Oxidation (M)
340	580.4500	1158.8854	1158.5993	0.2862	1	33	1.9	1	R.TREEIQEVR.S
367	597.3700	1192.7254	1192.5580	0.1674	0	86	7.3e-06	1	R.VDGMDILCVR.E + Carbamidomethyl (C); Oxidation (M)
413	669.3000	1336.5854	1335.6857	0.8998	0	78	4.9e-05	1	K.GPILMELQTYR.Y + Oxidation (M)
434	706.4100	1410.8054	1410.7719	0.0336	0	92	2e-06	1	R.LEEGPPVTTVLTR.E
438	718.3500	1434.6854	1434.6748	0.0107	0	51	0.021	1	K.LPCIFICENNR.Y + 2 Carbamidomethyl (C)
442	725.3700	1448.7254	1448.7181	0.0074	0	103	1.4e-07	1	R.MVNSNLASVEELK.E + Oxidation (M)
465	804.9600	1607.9054	1607.8341	0.0713	1	18	42	1	R.SGKGPILMELQTYR.Y + Oxidation (M)

- *Protein sequence with matched peptidesin bold red:*

1 MRGSFANDAT FEIKKCDLHR **LEEGPPVTTV LTR**EDGLKYY R**MMQTVR**RME

51 LKADQLYKQK IIRGFCHLCD GQEACCVGLE AGINPTDHLI TAYR**AHGFTF**

101 **TR**GLSVR**EIL AELTGR**KGGC AKGKGGSMHM YAKNFYGGNG IVGAQVPLGA

151 GIALACKYNG KDEVCLTLYG DGAANQGQIF EAYNMAALWK **LPCIFICENN**

201 **RYGMGTSVER AAASTDYYKR GDFIPGLRVD GMDILCVR**EA TRFAAAYCR**S**

251 **GKGPILMELQ TYR**YHGHEMS DPGVSYR**TRE EIQEVRSKSD PIMLLK**DR**MV**

301 **NSNLASVEEL K**EIDVEVRKE IEDAAQFATA DPEPPLEELG YHIYSSDPPF

351 EVRGANQWIK FKSVS

Methionine adenosyltransferase 2 beta (2-DE batch: 2090; rank 6):

- *List of tryptic peptides recovered:*

Query	Observed	Mr(expt)	Mr(calc)	Delta	Miss	Score	Expect	Rank	Peptide
143	361.3200	720.6254	721.3759	-0.7504	0	14	1.4e+02	1	K.VQFSNK.S
146	364.0900	726.1654	727.4017	-1.2363	0	21	23	5	R.FPTHVK.D
179	395.8900	789.7654	789.4232	0.3422	1	27	6.8	2	K.TKLDGEK.A
187	410.4300	818.8454	818.4208	0.4247	0	27	7.8	2	R.MLDPSIK.G + Oxidation (M)
188	410.6600	819.3054	819.3909	-0.0855	0	33	1.9	1	K.DVATVCR.Q + Carbamidomethyl (C)
242	468.7300	935.4454	934.4178	1.0276	0	29	4.8	1	R.NAQLDCSK.L + Carbamidomethyl (C)
251	494.2000	986.3854	985.5556	0.8298	0	45	0.091	1	K.LETLGIGQR.T
318	578.9900	1155.9654	1155.6976	0.2679	0	66	0.00086	1	R.VLVTGATGLLGR.A
319	580.9900	1159.9654	1159.6488	0.3166	0	35	1.1	1	R.IPILYGEVEK.L
389	656.3300	1310.6454	1310.6830	-0.0376	0	46	0.069	1	R.LVEEEVNIPNR.R
406	670.4300	1338.8454	1338.7619	0.0835	0	84	1.1e-05	1	K.AVLENNLGAAVLR.I
419	692.8400	1383.6654	1383.6592	0.0063	0	54	0.014	1	K.LEESAVTVMFDK.V + Oxidation (M)
440	730.3400	1458.6654	1457.7402	0.9253	0	38	0.47	1	R.EEDIPAPLNLYGK.T
250	490.3900	1468.1482	1466.7841	1.3641	1	54	0.014	1	R.LVEEEVNIPNRR.V
486	952.1100	1902.2054	1901.9629	0.2426	1	71	0.00018	1	R.NAQLDCSKLETLGIGQR.T + Carbamidomethyl (C)
376	635.2700	1902.7882	1902.9469	-0.1587	1	(24)	11	1	R.NAQLDCSKLETLGIGQR.T + Carboxymethyl (C)
477	870.4100	2608.2082	2607.2888	0.9193	0	59	0.0042	1	R.RPDVVENQPDAASQLNVDASGNLAK.E

- *Protein sequence with matched peptidesin bold red:*

```
1 MVGREKELSI HFVPGSCRLV EEEVNIPNRR VLVTGATGLL GRAVHKEFQQ
51 NNWHAVGCGF RRARPKFEQV NLLDSNAVHH IIHDFQPHVI VHCAAERRPD
101 VVENQPDAAS QLNVDASGNL AKEAAAVGAF LIYISSDYVF DGTNPPYREE
151 DIPAPLNLYG KTKLDGEKAV LENNLGAAVL RIPILYGEVE KLEESAVTVM
201 FDKVQFSNKS ANMDHWQQRF PTHVKDVATV CRQLAEKRML DPSIKGTFHW
251 SGNEQMTKYE MACAIADAFN LPSSHLRPIT DSPVLGAQRP RNAQLDCSKL
301 ETLGIGQRTP FRIGIKESLW PFLIDKRWRQ TVFH
```

Proteasome subunit beta-2 (2-DE batch: 2800; Rank 7):

- *List of tryptic peptides recovered:*

Query	Observed	Mr(expt)	Mr(calc)	Delta	Miss	Score	Expect	Rank	Peptide
130	382.7100	763.4054	763.4228	-0.0174	0	21	30	1	K.NVQLYK.M
226	500.8500	999.6854	999.5025	0.1829	0	19	41	1	R.YYTPTISR.E
230	509.0100	1016.0054	1016.5185	-0.5131	1	28	5.9	1	R.RNLADCLR.S + Carbamidomethyl (C)
269	539.0300	1076.0454	1075.5696	0.4759	0	74	0.00015	1	R.VAASNIVQMK.D + Oxidation (M)
343	653.8500	1305.6854	1305.7445	-0.0591	0	38	0.52	1	R.FILNLPTFSVR.I
391	735.4000	1468.7854	1468.7310	0.0544	0	35	0.83	1	K.NGIHDLDNISFPK.Q
421	806.4900	1610.9654	1610.7689	0.1966	0	56	0.0069	1	R.NGYELSPTAAANFTR.R
283	563.1500	1686.4282	1685.8043	0.6239	1	29	4.2	1	R.VAASNIVQMKDDHDK.M + Oxidation (M)
337	647.0400	1938.0982	1938.0211	0.0771	1	53	0.016	1	R.IIDKNGIHDLDNISFPK.Q

- *Protein sequence with matched peptidesin bold red:*

```
1 MEYLIGIQGP DYVLVASDRV AASNIVQMKD DHDKMFKMSE KILLLCVGEA
51 GDTVQFAEYI QKNVQLYKMR NGYELSPTAA ANFTRRNLAD CLRSRTPYHV
101 NLLLAGYDEH EGPALYYMDY LAALAKAPFA AHGYGAFLTL SILDRYYTPT
151 ISRERAVELL RKCLEELQKR FILNLPTFSV RIIDKNGIHD LDNISFPKQG
201 S
```

Cat eye syndrome critical region protein 5 isoform 2 (2-DE batch: 4081; rank 8):

- *List of tryptic peptides recovered:*

Query	Observed	Mr(expt)	Mr(calc)	Delta	Miss	Score	Expect	Rank	Peptide
172	507.7000	1013.3854	1013.5618	-0.1763	0	49	0.038	1	R.LVNSQGQLR.V
188	523.0500	1044.0854	1043.5611	0.5243	0	35	1.2	1	R.NVVTVDELR.M
189	527.0500	1052.0854	1051.4974	0.5880	0	29	4.3	1	K.LFSEYHEK.R
102	403.6200	1207.8382	1207.5985	0.2396	1	14	1.5e+02	4	K.LFSEYHEKR.M
271	647.8600	1293.7054	1293.7656	-0.0602	0	66	0.00079	1	R.IEGVLLLGEPVR.W
292	705.3200	1408.6254	1408.6695	-0.0441	0	37	0.57	1	K.ATHDGAPELGAGGTR.Q
162	470.6100	1408.8082	1408.6695	0.1387	0	(35)	0.92	1	K.ATHDGAPELGAGGTR.Q
347	783.4500	1564.8854	1564.7629	0.1225	0	(37)	0.53	1	R.MAFPLLDMVDLER.R + Oxidation (M)
354	791.4400	1580.8654	1580.7578	0.1076	0	62	0.0016	1	R.MAFPLLDMVDLER.R + 2 Oxidation (M)
423	911.3500	1820.6854	1822.0101	-1.3247	0	30	3	1	R.VPVVFVTNAGNILQHSK.A
282	675.3500	2023.0282	2021.9663	1.0619	0	(8)	5.4e+02	6	R.MLVSGQGPVMENAQGLGFR.N + 2 Oxidation (M)
463	1012.5300	2023.0454	2021.9663	1.0792	0	83	1.3e-05	1	R.MLVSGQGPVMENAQGLGFR.N + 2 Oxidation (M)
291	704.9800	2111.9182	2112.0137	-0.0955	0	36	0.77	1	R.NPQSTEPVLGGGEPPFHGHR.D
381	826.4200	2476.2382	2475.2355	1.0027	0	59	0.0035	1	R.YEGLMGKPSILTYQYAEDLIR.R + Oxidation (M)
421	910.2200	2727.6382	2727.3095	0.3287	0	56	0.0078	1	K.AQELSALLGCEVDADQVILSHSPMK.L + Carboxymethyl (C); Oxidation (M)

- *Protein sequence with matched peptidesin bold red:*

```
1 MAAWGCVAAL GAARGLCWRA ARAAAGLQGR PARRCYAVGP AQSPPTFGFL
51 LDINGVLVRG HRVIPAALKA FRRLVNSQGQ LRVPVVFVTN AGNILQHSKA
101 QELSALLGCE VDADQVILSH SPMKLFSEYH EKRMLVSGQG PVMENAQGLG
151 FRNVVTVDEL RMAFPLLDMV DLERRLKTTP LPRNDFPRIE GVLLLGEPVR
201 WETSLQLIMD VLLSNGSPGA GLATPPYPHL PVLASNMDLL WMAEAKMPRF
251 GHGTFLLCLE TIYQKVTGKE LRYEGLMGKP SILTYQYAED LIRRQAERRG
301 WAAPIRKLYA VGDNPMSDVY GANLFHQYLQ KATHDGAPEL GAGGTRQQQP
351 SASQSCISIL VCTGVYNPRN PQSTEPVLGG GEPPFHGHRD LCFSPGLMEA
401 SHVVNDVNEA VQLVFRKEGW ALE
```

Casein kinase 2 alpha (2-DE batch: 3121; rank 9-1):

- *List of tryptic peptides recovered:*

Query	Observed	Mr(expt)	Mr(calc)	Delta	Miss	Score	Expect	Rank	Peptide
95	379.2100	756.4054	756.4494	-0.0439	0	22	23	3	K.ILENLR.G
289	655.8100	1309.6054	1309.7605	-0.1551	0	45	0.1	1	R.GGPNIITLADIVK.D
302	679.3700	1356.7254	1356.6898	0.0356	0	12	1.9e+02	2	R.VYTDVNTHRPR.E
413	866.5000	1730.9854	1730.8628	0.1227	0	68	0.00041	1	R.TPALVFEHVNNTDFK.Q
232	578.1200	1731.3382	1730.8628	0.4754	0	(29)	4.1	1	R.TPALVFEHVNNTDFK.Q
420	885.8100	1769.6054	1769.8472	-0.2417	0	60	0.0026	1	K.YSEVFEAINITNNEK.V
437	932.9000	1863.7854	1864.0418	-0.2564	1	67	0.00057	1	R.GGPNIITLADIVKDPVSR.T
268	622.6900	1865.0482	1864.0418	1.0064	1	(53)	0.015	1	R.GGPNIITLADIVKDPVSR.T
271	625.6000	1873.7782	1872.8544	0.9238	0	33	1.5	1	K.EPFFHGHDNYDQLVR.I
362	776.0400	2325.0982	2324.1437	0.9545	0	15	96	1	R.FVHSENQHLVSPEALDFLDK.L

- *Protein sequence with matched peptidesin bold red:*

1 MSGPVPSRAR **VYTDVNTHRP R**EYWDYESHV VEWGNQDDYQ LVRKLGRGK**Y**
51 **SEVFEAINIT NNEK**VVVKIL KPVKKKKIKR EIK**ILENLRG GPNIITLADI**
101 **VKDPVSRTPA LVFEHVNNTD FK**QLYQTLTD YDIRFYMYEI LKALDYCHSM
151 GIMHRDVKPH NVMIDHEHRK LRLIDWGLAE FYHPGQEYNV RVASRYFKGP
201 ELLVDYQMYD YSLDMWSLGC MLASMIFRK**E PFFHGHDNYD QLVR**IAKVLG
251 TEDLYDYIDK YNIELDPRFN DILGRHSRKR WER**FVHSENQ HLVSPEALDF**
301 **LDK**LLRYDHQ SRLTAREAME HPYFYTVVKD QARMGSSSMP GGSTPVSSAN
351 MMSGISSVPT PSPLGPLAGS PVIAAANPLG MPVPAAAGAQ Q

MLL septin like fusion protein MSF-B (2-DE batch: 3121; rank 9-2):

- *List of tryptic peptides recovered:*

Query	Observed	Mr(expt)	Mr(calc)	Delta	Miss	Score	Expect	Rank	Peptide
189	516.6500	1031.2854	1031.4883	-0.2029	0	35	1	1	K.SVQPTSEER.I
192	518.4200	1034.8254	1035.5964	-0.7710	0	34	1.3	1	K.STLINTLFK.S
206	526.6600	1051.3054	1050.6801	0.6253	0	6	8.8e+02	7	K.VVNIVPVIAK.A
209	528.3800	1054.7454	1055.4924	-0.7469	0	25	9.1	1	K.FINDQYEK.Y
80	357.9500	1070.8282	1070.5244	0.3038	0	(14)	1.4e+02	4	K.SITHDIEEK.G
218	536.5300	1071.0454	1070.5244	0.5211	0	37	0.69	1	K.SITHDIEEK.G
230	570.7600	1139.5054	1140.4207	-0.9153	0	54	0.014	1	K.EFDEDSEDR.L
276	639.2500	1276.4854	1276.6411	-0.1557	0	49	0.039	1	K.YLQEEVNINR.K
313	703.4400	1404.8654	1404.7361	0.1294	1	23	16	1	K.YLQEEVNINRK.K
333	719.8700	1437.7254	1437.6888	0.0366	0	29	4.1	1	K.DITSSIHFEAYR.V
363	776.4000	1550.7854	1550.6882	0.0972	0	66	0.0008	1	R.LNEGSSAMANGVEEK.E + Oxidation (M)
231	575.7200	1724.1382	1723.7537	0.3845	1	34	1.3	1	K.EFDEDSEDRLVNEK.F
416	873.6800	1745.3454	1745.9200	-0.5745	0	43	0.16	1	R.ITADLLSNGIDVYPQK.E
466	1013.6100	2025.2054	2024.9877	0.2177	0	45	0.07	1	K.APVDFGYVGIDSILEQMR.R + Oxidation (M)
366	784.5400	2350.5982	2349.9940	0.6042	1	25	11	1	R.LNEGSSAMANGVEEKEPEAPEM.- + 2 Oxidation (M)

- *Protein sequence with matched peptidesin bold red:*

1 MEPPASKVPE VPTAPATDAA PKRVEIQMPK PAEAPTAPSP AQTLENSEPA
51 PVSQLQSRLE PKPQPPVAEA TPRSQEATEA APSCVGDMAD TPRDAGLKQA
101 PASRNEK**APV DFGYVGIDSI LEQMR**RKAMK QGFEFNIMVV GQSGLGK**STL**
151 **INTLFK**SKIS RK**SVQPTSEE R**IPKTIEIK**S ITHDIEEK**GV RMKLTVIDTP
201 GFGDHINNEN CWQPIMK**FIN DQYEKYLQEE VNINRK**KRIP DTRVHCCLYF
251 IPATGHSLRP LDIEFMKRLS K**VVNIVPVIA K**ADTLTLEER VHFKQR**ITAD**
301 **LLSNGIDVYP QKEFDEDSED RLVNEK**FREM IPFAVVGSDH EYQVNGKRIL
351 GRKTKWGTIE VENTTHCEFA YLRDLLIRTH MQNIK**DITSS IHFEAYR**VKR
401 **LNEGSSAMAN GVEEKEPEAP EM**

Heterogeneous nuclear ribonucleoproteins A2 (2-DE batch: 2056; rank 10):

- *List of tryptic peptides recovered:*

Query	Observed	Mr(expt)	Mr(calc)	Delta	Miss	Score	Expect	Rank	Peptide
57	419.0400	836.0654	835.4188	0.6467	1	24	18	4	R.EKEQFR.K
79	457.3200	912.6254	911.3733	1.2521	0	34	1.4	1	R.GGSDGYGSGR.G
111	506.0800	1010.1454	1009.4573	0.6882	0	22	21	1	K.LTDCVVMR.D + Carboxymethyl (C); Oxidation (M)
112	507.6100	1013.2054	1012.4363	0.7692	0	43	0.17	1	R.GGNFGFGDSR.G
120	526.0500	1050.0854	1049.4342	0.6513	0	28	6	1	R.DYFEEYGK.I
145	583.4200	1164.8254	1164.5159	0.3095	0	41	0.28	1	K.EDTEEHHLR.D
162	610.8100	1219.6054	1220.5455	-0.9401	0	44	0.15	1	R.QEMQEVQSSR.S
170	619.7700	1237.5254	1236.5405	0.9850	0	(27)	6.2	1	R.QEMQEVQSSR.S + Oxidation (M)
199	669.8600	1337.7054	1337.6939	0.0115	0	43	0.15	1	R.EESGKPGAHVTVK.K
209	689.7900	1377.5654	1376.6222	0.9433	0	35	0.92	1	R.GGGGNFGPGPGSNFR.G
218	705.7600	1409.5054	1409.6800	-0.1745	0	42	0.19	1	K.YHTINGHNAEVR.K
335	849.0000	1695.9854	1694.7577	1.2277	0	58	0.0043	1	R.GFGFVTFDDHDPVDK.I
388	900.0500	1798.0854	1797.9149	0.1706	0	71	0.0002	1	K.LFIGGLSFETTEESLR.N
174	627.3900	1879.1482	1878.9588	0.1894	1	57	0.0054	1	K.LFVGGIKEDTEEHHLR.D
488	1103.2900	2204.5654	2204.8930	-0.3276	0	56	0.0066	1	R.NMGGPYGGGNYGPGGSGGSGGYGGR.S + Oxidation (M)
244	736.1000	2205.2782	2204.8930	0.3851	0	(35)	1	1	R.NMGGPYGGGNYGPGGSGGSGGYGGR.S + Oxidation (M)

- *Protein sequence with matched peptidesin bold red:*

1 MER**EKEQFR**K **LFIGGLSFET TEESLR**NYYE QWGK**LTDCVV MR**DPASKRSR
51 GFGFVTFSSM AEVDAAMAAR PHSIDGRVVE PKRAVAR**EES GKPGAHVTVK**
101 K**LFVGGIKED TEEHHLRDYF EEYGK**IDTIE IITDRQSGKK R**GFGFVTFDD**
151 **HDPVDK**IVLQ K**YHTINGHNA EVR**KALSR**QE MQEVQSSR**SG R**GGNFGFGDS**
201 **RGGGGNFGPG PGSNFRGGSD GYGSGR**GFGD GYNGYGGGPG GGNFGGSPGY
251 GGGRGGYGGG GPGYGNQGGG YGGGYDNYGG GNYGSGNYND FGNYNQQPSN
301 YGPMKSGNFG GSR**NMGGPYG GGNYGPGGSG GSGGYGGR**SR Y

Chain A, Isovaleryl-Coa Dehydrogenase (2-DE batch: 3120; rank 11-1):

- *List of tryptic peptides recovered:*

Query	Observed	Mr(expt)	Mr(calc)	Delta	Miss	Score	Expect	Rank	Peptide
81	366.1400	730.2654	730.3609	-0.0955	0	29	4.7	1	K.AQEIDR.S
147	460.6800	919.3454	918.3575	0.9879	0	24	14	1	K.MADMYTR.L + 2 Oxidation (M)
172	493.1800	984.3454	983.5076	0.8378	0	42	0.19	1	R.QYVYNVAK.A
174	500.6600	999.3054	999.5349	-0.2294	0	34	1.3	1	K.TDLAAVPASR.G
209	542.0700	1082.1254	1081.5920	0.5334	0	45	0.11	1	K.FLQEHLAPK.A
220	565.1300	1128.2454	1128.5676	-0.3221	0	19	44	1	K.GNHYILNGNK.F
226	575.3800	1148.7454	1147.4386	1.3068	0	26	7.5	1	K.ACDEGHCTAK.D + 2 Carbamidomethyl (C)
101	392.1700	1173.4882	1173.5965	-0.1083	0	(14)	1.5e+02	5	K.IGHFQLMQGK.M + Oxidation (M)
231	588.3800	1174.7454	1173.5965	1.1490	0	20	30	1	K.IGHFQLMQGK.M + Oxidation (M)
267	639.3200	1276.6254	1275.6935	0.9320	0	35	1.1	1	K.IPAANILGHENK.G
274	647.7800	1293.5454	1293.6565	-0.1110	0	57	0.0058	1	K.LYEIGAGTSEVR.R
287	668.7300	1335.4454	1335.6419	-0.1964	1	18	46	1	K.AQEIDRSNEFK.N
324	725.9100	1449.8054	1449.7576	0.0479	1	18	46	1	K.LYEIGAGTSEVRR.L
326	726.4500	1450.8854	1450.7490	0.1365	0	(16)	76	1	K.GVYVLMSGLDLER.L
331	734.3800	1466.7454	1466.7439	0.0015	0	75	9.6e-05	1	K.GVYVLMSGLDLER.L + Oxidation (M)
363	786.8500	1571.6854	1571.6596	0.0259	0	69	0.00041	1	R.GSNTCELIFEDCK.I + 2 Carbamidomethyl (C)
466	997.0000	1991.9854	1991.9912	-0.0058	0	71	0.00021	1	-.HSLLPVDDAINGLSEEQR.Q
281	665.3300	1992.9682	1991.9912	0.9770	0	(43)	0.16	1	-.HSLLPVDDAINGLSEEQR.Q
429	900.5600	2698.6582	2698.4993	0.1589	0	41	0.2	1	R.LVLAGGPLGLMQAVLDHTIPYLHVR.E + Oxidation (M)

- *Protein sequence with matched peptidesin bold red:*

1 **HSLLPVDDAI NGLSEEQR**QL RQTMAK**FLQE HLAPKAQEID RSNEFK**NLRE
51 FWKQLGNLGV LGITAPVQYG GSGLGYLEHV LVMEEISRAS GAVGLSYGAH
101 SNLCINQLVR NGNEAQKEKY LPKLISGEYI GALAMSEPNA GSDVVSMKLK
151 AEKK**GNHYIL NGNK**FWITNG PDADVLIVYA K**TDLAAVPAS R**GITAFIVEK
201 GMPGFSTSKK LDKLGMR**GSN TCELIFEDCK IPAANILGHE NKGVYVLMSG**
251 **LDLERLVLAG GPLGLMQAVL DHTIPYLHVR** EAFGQK**IGHF QLMQGKMADM**
301 **YTR**LMACR**QY VYNVAKACDE GHCTAK**DCAG VILYSAECAT QVALDGIQCF
351 GGNGYINDFP MGRFLRDAK**L YEIGAGTSEV RR**LVIGRAFN ADFH

Fructose bisphosphate aldolase (2-DE batch: 3120; rank 11-2):

- *List of tryptic peptides recovered:*

Query	Observed	Mr(expt)	Mr(calc)	Delta	Miss	Score	Expect	Rank	Peptide
210	529.5100	1057.0054	1056.5564	0.4491	0	66	0.00089	1	R.AEVNGLAAQGK.Y
258	606.7100	1211.4054	1212.6575	-1.2520	1	34	1.2	1	K.RAEVNGLAAQGK.Y
389	827.0100	1652.0054	1651.9257	0.0798	0	71	0.00024	1	R.TPSALAILENANVLAR.Y
491	1123.1000	2244.1854	2242.9767	1.2088	0	51	0.018	1	K.YEGSGEDGGAAAQSLYIANHAY.-
465	1007.9000	3020.6782	3020.4801	0.1981	0	22	14	1	R.YASICQQNGIVPIVEPEILPDGDHDLK.R + Carboxymethyl (C)

- *Protein sequence with matched peptidesin bold red:*

1 MPHSYPALSA EQKKELSDIA LRIVAPGKGI LAADESVGSM AKRLSQIGVE
51 NTEENRRLYR QVLFSADDRV KKCIGGVIFF HETLYQKDDN GVPFVRTIQD
101 KGIVVGIKVD KGVVPLAGTD GETTTQGLDG LSERCAQYKK DGADFAKWRC
151 VLKISER**TPS ALAILENANV LARYASICQQ NGIVPIVEPE ILPDGDHDLK**
201 RCQYVTEKVL AAVYKALSDH HVYLEGTLLK PNMVTPGHAC PIKYTPEEIA
251 MATVTALRRT VPPAVPGVTF LSGGQSEEEA SFNLNAINRC PLPRPWALTF
301 SYGRALQASA VNAWRGQRDN AGAATEEFIK **RAEVNGLAAQ GKYEGSGEDG**
351 **GAAAQSLYIA NHAY**

Proteasome subunit p42 (2-DE batch: 3120; rank 11-3):

- *List of tryptic peptides recovered:*

Query	Observed	Mr(expt)	Mr(calc)	Delta	Miss	Score	Expect	Rank	Peptide
162	473.8100	945.6054	945.4014	0.2040	0	23	16	1	R.EMFNYAR.D + Oxidation (M)
167	484.7700	967.5254	968.4199	-0.8945	0	48	0.056	1	R.FSEGTSADR.E
175	505.0700	1008.1254	1007.5036	0.6219	1	47	0.07	1	R.DKALQDYR.K
266	637.2600	1272.5054	1272.6350	-0.1295	0	54	0.011	1	K.HGEIDYEAIVK.L
277	653.5400	1305.0654	1304.6837	0.3818	0	17	64	1	K.IHIDLPNEQAR.L
298	683.0600	1364.1054	1364.6758	-0.5704	0	24	14	1	R.AVASQLDCNFLK.V + Carbamidomethyl (C)
304	693.4700	1384.9254	1383.6275	1.2979	0	29	4.3	1	R.NVCTEAGMFAIR.A + Carbamidomethyl (C); Oxidation (M)
309	698.7900	1395.5654	1395.7102	-0.1447	0	50	0.032	1	R.VALDMTTLTIMR.Y + 2 Oxidation (M)
321	720.9100	1439.8054	1439.8348	-0.0293	0	48	0.044	1	K.ALQSVGQIVGEVLK.Q
428	899.5100	1797.0054	1796.9672	0.0382	0	25	8.6	1	R.EVIELPLTNPELFQR.V
311	701.5300	2101.5682	2102.9401	-1.3720	0	32	2.1	1	R.DHQPCIIFMDEIDAIGGR.R + Carboxymethyl (C); Oxidation (M)

- *Protein sequence with matched peptidesin bold red:*

1 MADPR**DKALQ DYR**KKLLEHK EIDGRLKELR EQLKELTKQY EKSENDLK**AL**
51 **QSVGQIVGEV LK**QLTEEKFI VKATNGPRYV VGCRRQLDKS KLKPGTR**VAL**
101 **DMTTLTIMR**Y LPREVDPLVY NMSHEDPGNV SYSEIGGLSE QIRELR**EVIE**
151 **LPLTNPELFQ R**VGIIPPKGC LLYGPPGTGK TLLAR**AVASQ LDCNFLK**VVS
201 SSIVDKYIGE SARLIR**EMFN YARDHQPCII FMDEIDAIGG RRFSEGTSAD**
251 **R**EIQRTLMEL LNQMDGFDTL HRVKMTMATN RPDTLDPALL RPGRLDRK**IH**
301 **IDLPNEQAR**L DILKIHAGPI TK**HGEIDYEA IVK**LSDGFNG ADLR**NVCTEA**
351 **GMFAIR**ADHD FVVQEDFMKA VRKVADSKKL ESKLDYKPV
